# The Eukaryotic Mismatch Recognition Complexes Track with the Replisome during DNA Synthesis

**DOI:** 10.1371/journal.pgen.1005719

**Published:** 2015-12-18

**Authors:** Joanna E. Haye, Alison E. Gammie

**Affiliations:** Department of Molecular Biology, Princeton University, Princeton, New Jersey, United States of America; Duke University, UNITED STATES

## Abstract

During replication, mismatch repair proteins recognize and repair mispaired bases that escape the proofreading activity of DNA polymerase. In this work, we tested the model that the eukaryotic mismatch recognition complex tracks with the advancing replisome. Using yeast, we examined the dynamics during replication of the leading strand polymerase Polε using Pol2 and the eukaryotic mismatch recognition complex using Msh2, the invariant protein involved in mismatch recognition. Specifically, we synchronized cells and processed samples using chromatin immunoprecipitation combined with custom DNA tiling arrays (ChIP-chip). The Polε signal was not detectable in G1, but was observed at active origins and replicating DNA throughout S-phase. The Polε signal provided the resolution to track origin firing timing and efficiencies as well as replisome progression rates. By detecting Polε and Msh2 dynamics within the same strain, we established that the mismatch recognition complex binds origins and spreads to adjacent regions with the replisome. In mismatch repair defective PCNA mutants, we observed that Msh2 binds to regions of replicating DNA, but the distribution and dynamics are altered, suggesting that PCNA is not the sole determinant for the mismatch recognition complex association with replicating regions, but may influence the dynamics of movement. Using biochemical and genomic methods, we provide evidence that both MutS complexes are in the vicinity of the replisome to efficiently repair the entire spectrum of mutations during replication. Our data supports the model that the proximity of MutSα/β to the replisome for the efficient repair of the newly synthesized strand before chromatin reassembles.

## Author Summary

During replication, errors that escape the replication machinery are identified and repaired by DNA mismatch repair proteins. A mismatch in the helix is recognized by MutS homologs and subsequent events include excision of the error-containing strand followed by re-synthesis. A critical step in this process is directing repair to the newly synthesized strand. Current data suggest that transient discontinuities in the DNA backbone, known as nicks, generated during replication serve as the strand discrimination signals. Additionally, proteins that package DNA have the capacity to block mismatch recognition and are known to rapidly assemble behind the replication fork. Thus, there must be a short window of opportunity for the mismatch recognition complexes to scan for mismatches and access the strand discrimination signals. To address these issues, we tested the model that the mismatch recognition complexes track with the replisome. We employed high resolution genomic methods to determine that during replication, the mismatch recognition complexes bind origins of replication and advances with the replisome. The findings support the hypothesis that the mismatch recognition proteins track with the DNA replication machinery to accurately survey and repair the newly synthesized strands while the DNA is unpackaged and strand specificity signals are accessible.

## Introduction

During cell division, accurate DNA replication is essential to preserve the integrity of the genome and defects in this process result in diseases including hereditary and sporadic cancers [1]. In eukaryotes, the replicative DNA polymerases, Polε and Polδ, perform leading and lagging strand synthesis respectively [2–5]. The proofreading function of the polymerases combined with the recognition and repair of mismatches ensures faithful transmission of genetic information during each round of replication. The errors generated during replication include single base mismatches, single nucleotide insertion/deletion loops (indels) at microsatellites (MS) [reviewed in 6]. Microsatellites are repeat regions of 1–10 bp repeat units, which frequently undergo expansion and contraction due to slippage of the polymerases during replication [7]. In prokaryotes, homodimeric MutS binds the full range of mismatches [reviewed in 6]. In eukaryotes, MutS complexes are heterodimers with differing mismatch recognition capabilities. MutSα (Msh2/Msh6) recognizes single base mismatches and single nucleotide indels at homopolymeric runs, and MutSβ (Msh2/Msh3) complex recognizes single nucleotide and larger indels [reviewed in 6]. MutSβ is also able to recognize certain base-base mismatches [8]. The ability of the mismatch repair (MMR) machinery to recognize the range of mismatches and target the newly synthesized, error-containing strand for repair is critical for maintaining fidelity during DNA replication.

The method of strand discrimination during mismatch repair in most prokaryotes and all eukaryotes appears to require discontinuities in the DNA backbone (nicks) and the replication sliding clamp, known as β clamp in prokaryotes or Proliferating Cell Nuclear Antigen, PCNA, in eukaryotes. *In vitro* experiments using cell extracts demonstrated that a nick is sufficient to direct repair to the strand containing the discontinuity [9, 10]. During DNA replication, the lagging strand has nicks ~200 bp apart [reviewed in 5]; whereas, the continuously synthesized leading strand may have long stretches without replication generated nicks [4]. However, during the replication process ribonucleotides (rNMP) are occasionally incorporated into the DNA molecule and are then cleaved by RNAase H2 [11–13], thereby increasing the density of nicks during synthesis [14, 15]. Because removal of RNAase H2 only causes a modest increase in mutation rates [14], it remains a possibility that the 3’-OH of the leading strand is the primary strand specificity signal. In addition to nicks, the replication sliding clamp has been implicated in strand discrimination. In eukaryotes, PCNA was shown to interact with MutSα/β mismatch recognition complexes [16–18]. It is postulated that the orientation specific association of PCNA with the DNA helix positions mismatch repair proteins to cleave the newly synthesized nicked strand rather than the template strand [19–22].

Taking into consideration the complex nature of the *in vivo* DNA environment during replication, it is important to note that the newly replicated DNA is thought to quickly re-assemble into nucleosomes behind the replisome [23] after which, a mismatch and the nicks are presumably less accessible to the MMR proteins. This potential for diminished accessibility is based on the fact that nucleosomes without replication/repair associated histone modifications [24] and other DNA bound proteins can block movement of MutS complexes along DNA [25, 26]. Taken together, the most efficient mechanism for detecting mismatches and for accessing the strand specificity signal would involve a close association between the mismatch recognition complexes and the replisome within the region where chromatin has been cleared.

Current data are consistent with the mismatch recognition complexes localizing to the replisome. Mass spectrometry analyses of human proteins at active replication forks, have identified MutS homologues [27]. In yeast, live cell-imaging demonstrated co-localization of MMR complexes and replisome components during S phase [28]. Additionally, a temporal coupling of MMR expression during S-phase and MMR efficiency has also been demonstrated [29]. Finally, as mentioned above, the eukaryotic and prokaryotic mismatch recognition proteins associate with the replication sliding clamps [30–32]. Taken together, the data support the model that the mismatch recognition proteins are associated with the replication machinery during S phase; however, whether the MMR recognition complexes track with the advancing replisome had not yet been demonstrated. The data presented in this work are consistent with the model that both MutSα and MutSβ track with the replisome during replication to efficiently scan protein-free DNA for the entire spectrum of errors and readily access the strand specificity signals in the form of proximal nicks in the DNA generated during replication.

## Results

### The dynamics of the leading strand polymerase during DNA synthesis are detectable using chromatin immunoprecipitation and tiling arrays

To determine if the mismatch recognition complexes track with the replisome, we first needed suitable controls to define the replication origins and to indicate the position of the advancing replisome during DNA replication. The minichromosome maintenance (Mcm) 2–7 helicase is a well-established predictor of potential origins of replication [33]. The Mcm 2–7 helicase is a component of pre-replication complexes that associate with origins during the G1 phase of the cell cycle [34]. We employed a hemagglutinin (HA) tagged Mcm4, a subunit of the replicative helicase, to indicate potential replication origins. Additionally, in a separate strain the leading strand polymerase served as the control for replisome progression. Specifically, we used a HA tagged version of Pol2, the catalytic subunit of Polε. We performed chromatin immunoprecipitations (ChIP) to detect what portions of the genome were associated with the replication proteins in G1 and throughout S-phase. All ChIP experiments included an untagged control for non-specific precipitation of certain DNA regions. This allowed for exclusion of regions of the genome that generate high background signal; for example, highly transcribed regions have a tendency to give a false positive signal in ChIP experiments [35].

Experiments were performed at 18°C to slow the replication process and improve the resolution of the signal for the advancing replisome. Initial arrest and progression through the cell cycle were monitored by determining the DNA content per cell using flow cytometry. One time point in G1 and six time points in S phase were subsequently processed for ChIP (S1 and S2 Figs). ChIP samples were labeled and hybridized to custom DNA tiling arrays. The arrays included 65 of the ~500 origins of replication in the yeast genome (S1 Table)[36, 37]. By measuring the peak corresponding to Mcm4 binding, we were able to mark the specific coordinates of the origins (S4 Fig).

**Fig 1:**
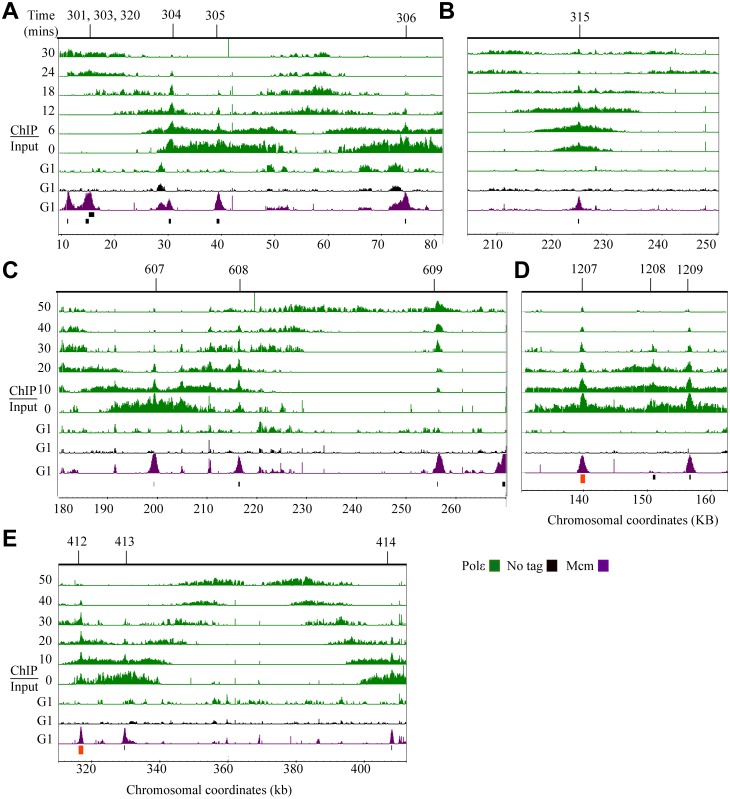
Polε dynamics during DNA replication. Analysis of Pol2 (Polε) dynamics during DNA replication using ChIP-chip. Each row corresponds to ChIP-chip signal at the indicated times at G1 or to the time point series taken during S phase (0 to 30 minutes or 0 to 50 minutes). The tiling array data were visualized using the Integrated Genome Browser program (Affymetrix) and are depicted as peaks correspond to log2 ratios (ChIP/Input). The y-axis is set to 2.5 (or a ~6-fold maximum signal). Black bars below the data denote position of origins in the genome databases. The red bars represent origins not found in the genome database. Chromosomal coordinates represent x 10^3^ kb. Mcm4 (Mcm) signal, shown in purple, is visible at potential origins during G1 and non-specific signals shown in black are detected in the no tag control IP during G1. Polε signal (green) is detected at active origins. Representative regions are shown including: **(A)** active origins (*ARS305* and *ARS306*) and adjacent inactive origins (*ARS301*, *ARS303*, *ARS304*and *ARS320*), **(B)** an early-efficient origin (*ARS315*), **(C)** adjacent early-efficient (*ARS607*), early-inefficient, (*ARS608*), late-inefficient, *ARS609*, **(D)** early-efficient origins (*ARS1207* and *ARS1209*) flanking an inactive origin (*ARS1208*), **(E)** a ~100 kb region of chromosome IV where the advancing forks converge.

We found that Mcm4 binding to potential origins is in agreement with previous studies and annotations (S1 Table). In addition, we showed that the highly reproducible Polε signal throughout S-phase functions as a good metric for replisome progression (Fig 1). Supplement S6 illustrates the reproducibility of the Mcm4 and Polε signals across multiple experimental trials. These data establish that chromatin immunoprecipitation in combination with DNA tiling arrays is an effective method for tracking the leading strand polymerase at origins of replication during DNA synthesis. A more detailed analysis of Polε progression during S-phase is presented in the following sections.

### After release from G1, Polε binding to active origins is consistent with known origin firing times

In G1 synchronized cells, all potential origins were detected by Mcm4 binding (Fig 1; S1 Table), consistent with previous studies [33]. We determined that the Polε signal appears at active origins only after release from G1. For example, *ARS301*, *ARS303* and *ARS320* are inactive origins [38] and are bound by Mcm4, but not by Polε (Fig 1A). In contrast, *ARS305* and *ARS306* are known active origins [39–41] and exhibit Mcm4 as well as Polε binding (Fig 1A); however, the Polε is evident only after release from G1. This finding is consistent previous studies using ChIP-PCR that detected Polε at origins during S-phase, but not at G1 of the cell cycle [34]. We find that the Polε signal appears at origins consistent with known firing times. Active origins of replication are known to fire at different times (early, middle and late) during S-phase [42]. A representative example of the differential timing is depicted in Fig 1C, where Polε binds the known late firing *ARS609* at a later time than it does the known early firing, adjacent origins *ARS607* and *ARS608* [36].

### Persistent Polε signal at certain origins may reflect an imprecision of firing times

The Polε signal typically diminishes at the origins after fork progression (Fig 1A and 1E); however, in some cases signal is observed at the origin at later time points (Fig 1B and 1D). One explanation for this signal is lack of synchrony. Alternatively, this may be a consequence of some replication origins firing with less precision during the cell cycle [reviewed in 42, 43, 44]. We favor the second explanation because there are examples from the same experiment where at certain origins the signal diminishes (e.g. at *ARS305* and *ARS306* in Fig 1A), suggesting synchrony, while at other origins the signal persists (e.g. *ARS315*, Fig 1B), consistent with less precision of firing of *ARS315* during the cell cycle.

### Polε signal intensity is consistent with origin firing efficiency

The Polε signal observed is also in agreement with the known differences in firing efficiency of each origin [36]. *ARS315* is highly efficient and fires in ~90% of each S-phase of the cell cycle [45]. In this study, *ARS315* exhibits a robust Polε signal initially localized that then migrates away from the origin over time (Fig 1B). Additionally, efficiently firing *ARS607* (fires >85% of the cell cycles) [46] displays a particularly robust Polε signal at the origin; whereas the adjacent, less efficient *ARS608* (fires in <10% of the cell cycles) [46] has a reduced signal (Fig 1C). Finally, highly efficient origins such as *ARS1207* and *ARS1209*, exhibit a strong Polε signal (Fig 1D).

### Polε signal advances bi-directionally away from origins with expected kinetics

We observed that the Polε signal throughout S-phase is consistent with the advancing replisome kinetics. Specifically, Polε signal first appears at the origins and advances bi-directionally to adjacent regions as the cells progress through S-phase of the cell cycle (Fig 1A–1E). The ~100 kb region on Chromosome IV represents a good example of Polε initially binding origins followed by the signal migrating to flanking regions up and downstream in subsequent time points (Fig 1E). By measuring the leading edges of the replisome signal, the average rate of replication fork progression was calculated as ~430 base pairs per minute (S2 Table). Previous studies showed that replication fork rate may vary with temperature, nutrient availability or drug treatment (summarized in Table 1). For example, experiments were performed at room temperature and replication fork rate was determined to be 1.6 kb/min, consistent with a faster doubling time [47]. In summary, Polε signal is detected at active origins with expected timing and firing efficiencies. Additionally, the movement of the Polε signal is consistent with the advancing replisome during S phase. These experiments established the foundation for a comparative analysis of the eukaryotic mismatch recognition complexes during S phase.

**Table 1:** Replisome progression rates.

Experiment	Rate kb/min	Reference
Proline medium	0.56	[87]
New DNA synthesis	2.9	[40]
New DNA synthesis	2.8	[39]
Psf2 subunit of GINS complex	1.6	[47]
New DNA synthesis in MMS	~0.45	[88]
Pol2 subunit of Polε at 18°C	~0.43	This study

### During replication, Msh2 binds origins and spreads to adjacent regions

**Fig 2:**
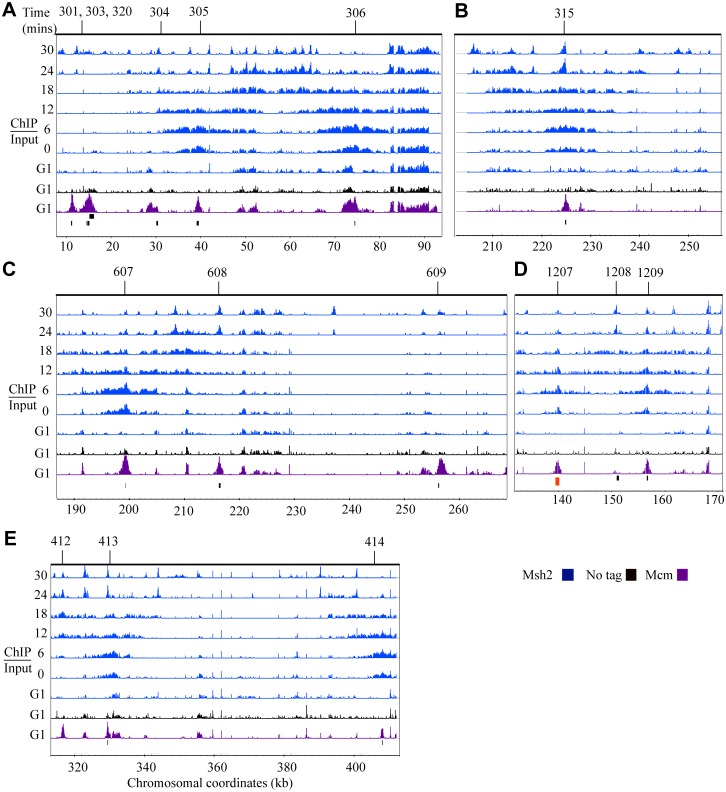
Msh2 dynamics during DNA replication. Time course ChIP-chip experiment for Msh2-myc (Msh2). Each row corresponds to ChIP-chip signal during G1 or to the time point series taken during S phase (0 to 30 minutes). The tiling array data were visualized using the Integrated Genome Browser program (Affymetrix) and are depicted as peaks correspond to log2 ratios (ChIP/Input). The y-axis is set to 2.5 (or a ~6-fold maximum signal). Black bars below the data denote position of origins in the genome databases. The red bars represent origins not found in the genome database. Chromosomal coordinates represent x 10^3^ kb. Mcm4 (Mcm) signal, shown in purple, is visible at potential origins during G1 and non-specific signals shown in black are detected in the no tag control IP during G1. Msh2 signal (blue) is detected at active origins. Representative regions are shown including: **(A)** active origins (*ARS305* and *ARS306*) and adjacent inactive origins (*ARS301*, *ARS303*, *ARS304* and *ARS320*), **(B)** an early-efficient origin (*ARS315*), **(C)** adjacent early-efficient (*ARS607*), early-inefficient, (*ARS608*), late-inefficient, *ARS609*, **(D)** early-efficient origins (*ARS1207* and *ARS1209*) flanking an inactive origin (*ARS1208*), **(E)** a ~100 kb region of chromosome IV where the advancing forks from *ARS413* and *ARS414* are observed.

With the appropriate controls for origin position and for replication fork migration established, we next aimed to determine the dynamics of the mismatch recognition complexes during S phase. MutSα (Msh2/Msh6) and MutSβ (Msh2/Msh3) are the two mismatch recognition complexes in eukaryotes that function in post-replicative mismatch repair [6, 48]. Since Msh2 is the invariable component of both complexes, we tagged Msh2 with the myc epitope, facilitating the detection of both MutSα and MutSβ complexes within the cell. We employed the methods described above to determine the mismatch recognition complex dynamics during S-phase and observed that the Msh2 signal is remarkably similar to Polε (compare Figs 1 and 2). Msh2 is observed at origins in S-phase, but not G1, and the signal progresses away from the origins bi-directionally (Fig 2A–2E). For example, the Msh2 signal originates at *ARS305* and *ARS306* and migrates bi-directionally from each origin (Fig 2A). Additionally, while Mcm4 signal is at *ARS301*, *ARS303*, *ARS304* and *ARS320* these inactive origins do not exhibit Msh2 signal as was observed for the Polε signal (Figs 1 and 2). Taken together, the data show that Msh2 binds and moves bi-directionally away from active origins (Mcm4 and Polε both bound), but does not bind inactive origins (Mcm4 only). It is important to note that we would not expect to detect significant signal of MutS complexes at mismatches because mismatches are rare within any given population of replicating cells [49–53]. The Msh2 signal in these experiments represents DNA surveillance during replication and not mismatch binding.

Similar to Polε (Fig 1), the time of appearance and intensity of the Msh2 signal is consistent with the expected timing and firing efficiencies of the origins (Fig 2). For example, the intensity and distribution of Msh2 signal at *ARS315* is consistent with the efficient, early firing of this origin (Fig 2B). In addition, for the early-efficient *ARS607* origin, the Msh2 signal is robust compared to the early-inefficient *ARS608* origin (Fig 2C). Correspondingly, the early-efficient *ARS1207* and *ARS1209* origins exhibit an early Msh2 signal that progressively migrates bi-directionally away from both origins while the *ARS1208* remains inactive (Fig 2D) as was observed for Polε (Fig 1). After fork progresses bi-directionally from the origins, some signal can be observed in the position of the origin. The absence of bi-directional movement from *ARS1208* is consistent with a previous study showing no Orc or Mcm4 binding at this origin [33].

We observed one potential difference between the Msh2 and Polε signal in regions behind the advancing replisome. While the Polε signal clears as the replisome advances (Fig 1), there is still some Msh2 signal in the region behind the replication fork (Fig 2). The persistent Msh2 signal is significantly higher than is observed in the “no tag” control. In the following section we discuss the significance of this persistent signal.

### Msh2 tracks with the replisome and persists transiently behind the advancing replication fork

Because the initial analyses showed similarities and differences in the dynamics of the Polε and Msh2 during S phase, we wanted to determine how precisely the signals coincided by using a strain tagged for both proteins. The G1 and S-phase samples from a doubly-tagged strain were processed as described above except that half the sample was processed for Msh2 ChIP and the other half for Polε ChIP.

**Fig 3:**
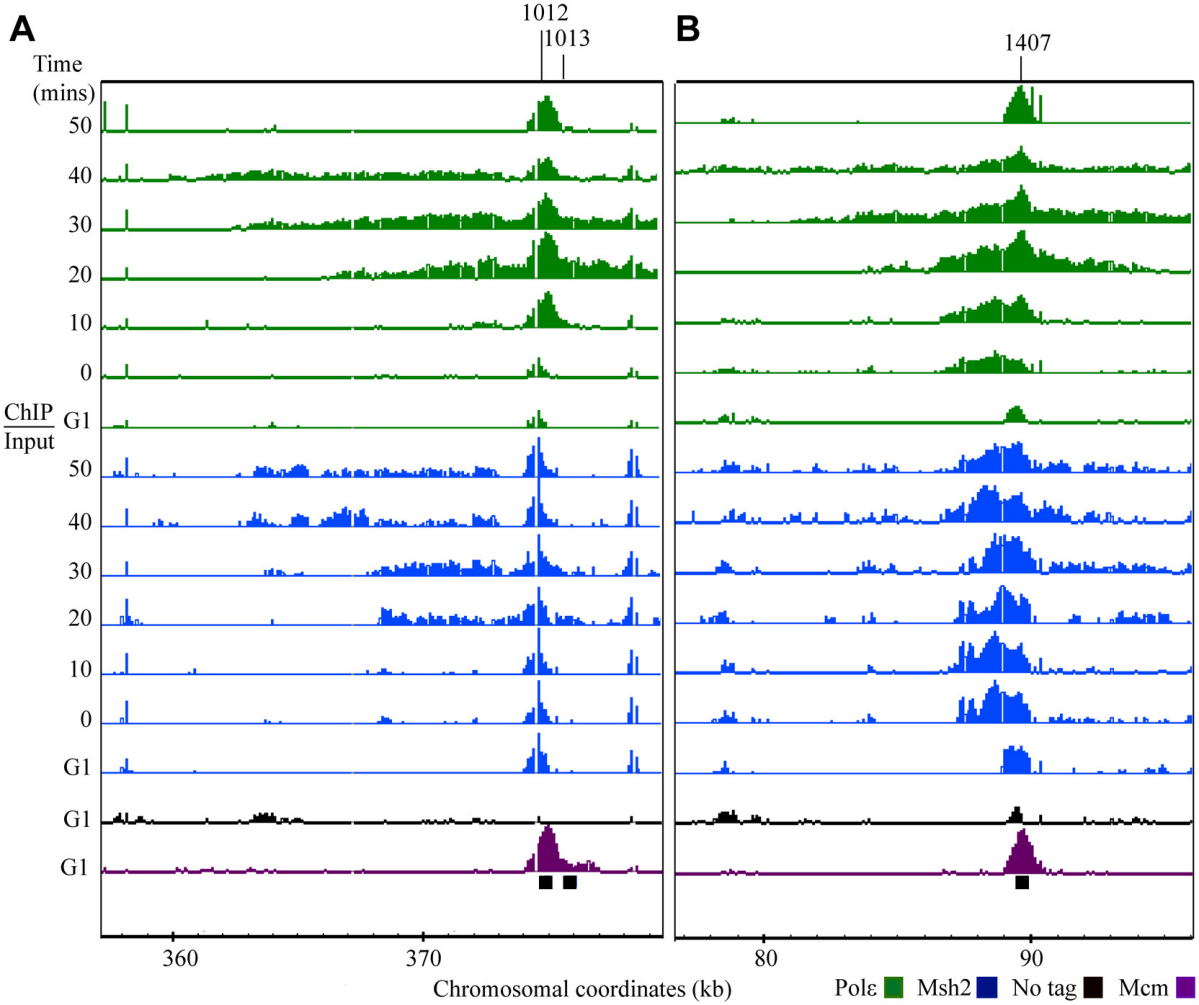
Msh2 and Polε dynamics are similar during DNA replication. Cells were fixed for 45 minutes, the samples were divided and ChIP was performed with specified antibodies to detect Polε-HA (green) and Msh2-myc (blue). The distribution was visualized using the Integrated Genome Browser program (Affymetrix) as log_2_ ratios (ChIP/Input) with the scale set at 2.5 (~ 6 fold increase) for all samples. Each row corresponds to ChIP-chip signal during G1 or to the time point series taken during S phase (0–50 min). Black bars below the data denote position of origins in the genome databases. Chromosomal coordinates represent x 10^3^ kb. Mcm4 (Mcm4) signal, shown in purple, is visible at potential origins during G1 and non-specific signals shown in black are detected in the no tag control IP during G1. Representative regions are shown including: **(A)** *ARS1407*, where there is an initial unidirectional distribution of signal that is followed by bi-directional progression at later time point, and **(B)** the early-efficient *ARS1012* and the early-inefficient *ARS1013*.

**Fig 4:**
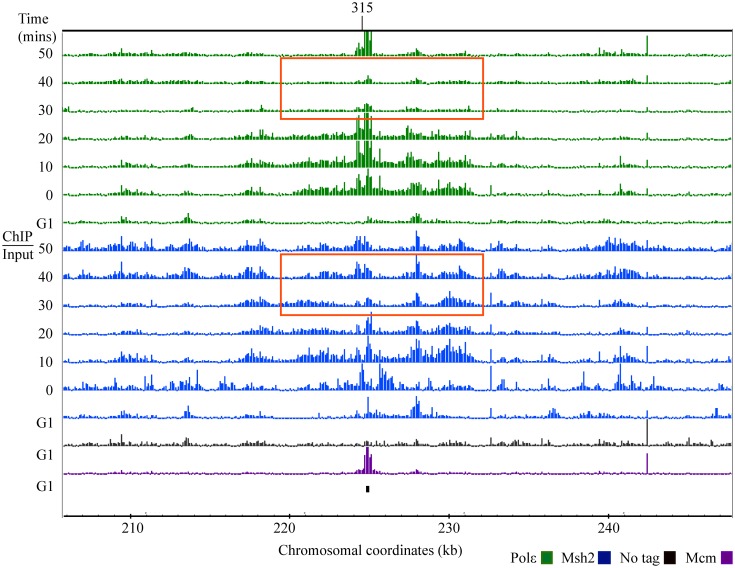
Msh2 persistence in the region behind the replisome. Cells were fixed for 45 minutes, the samples were divided and ChIP was performed with specified antibodies to detect Polε-HA (green) and Msh2-myc (blue). The distribution was visualized using the Integrated Genome Browser program (Affymetrix) as log_2_ ratios (ChIP/Input) with the scale set at 2.5 (~ 6 fold increase) for all samples. Each row corresponds to ChIP-chip signal during G1 or to the time point series taken during S phase (0–50 min). The black bar below the data denotes the position of *ARS315*. Chromosomal coordinates represent x 10^3^ kb. Mcm4 (Mcm) signal, shown in purple, is visible at potential origins during G1 and non-specific signals shown in black are detected in the no tag control IP during G1 The time points and region surrounding *ARS315* with a persistent Msh2 ChIP signal is indicated with red rectangles.

We examined the occupancy of both Polε and Msh2 at all of the 65 origins on the tiling arrays. Representative images of *ARS1012*, *ARS1013* and *ARS1407* are shown in Fig 3. The Msh2 signal is in regions occupied by Polε at each corresponding time point (Fig 3). These data are consistent with the mismatch recognition complexes loading at origins with a timing similar to the leading strand polymerase and associating with the replisome throughout DNA replication. Interestingly, there is a persistent Msh2 signal localized in the region behind the advancing replisome at *ARS315* (Fig 4). When examining 12 early firing origins, the Msh2 signals persist after Polε signal diminishes for 9 origins. The no tag control does not exhibit the signal and statistical analyses of replicates discussed below confirm that this persistent signal is significant.

**Fig 5:**
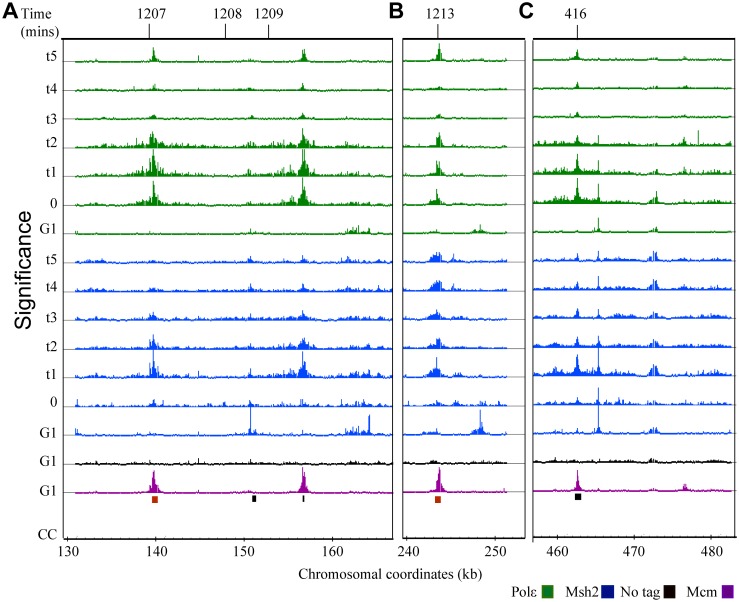
Msh2 and Polε exhibit co-incident signal during S phase. Three independently performed experiments were used to calculate *p*-values. The samples were analyzed at the time of arrest (G1) and six additional time points during S phase for Polε and Msh2. The data are visualized as the negative of the log_10_ of the calculated *p-*values using the Integrated Genome Browser for Mcm4 (Mcm, purple), no tag (black), Msh2 (blue) and Pol2 (Polε, green). Because the Mcm4 signal is so significant, the histogram is scaled to 27 (or reflecting a *p-*value ~10^−27^ for the most signal values). The Msh2 and the “no tag” control graphs are set to 10 and the Polε graphs to 20. Black bars below the data denote position of origins in the genome databases. The red bars represent origins not found in the genome database. Chromosomal coordinates represent x10^3^ kb. Representative regions are shown including: **(A)** early-efficient origins (*ARS1207* and *ARS1209*) flanking an inactive origin (*ARS1208*), **(B)** *ARS1213*, and **(C)** *ARS416*.

To determine the statistical significance of the co-incident signals of Msh2 and Polε, we employed Chipper Software [54] to assign *p*-values to the ChIP signals from the tiling arrays. We averaged three replicates for Mcm4, Polε, Msh2 and the no tag control. The data are visualized as the negative of the log_10_ of the calculated *p-*values (Fig 5A–5C). We examined the significance of the ChIP signals for all potential origins represented on the tiling arrays. Of the 65 putative origins on the arrays, 60 exhibited Mcm4 signal (*p* values ranged from 10^−5^ to 10^−40^). These 60 potential origins were used to calculate the occupancy by Polε and Msh2. A total of 55 origins (~91%) showed Polε and Msh2 signal during S-phase. Origins where no Polε or Msh2 signal is detected are origins that are not bound by Mcm4 or have previously been established as inefficient and firing only in a small percentage of each round of replication [39, 40, 46]. Importantly, the co-occupancy signal of Polε and Msh2 at origins and adjacent regions during replication is highly significant. The Polε *p* values ranged from 10^−3^ to 10^−25^ and Msh2 *p* values were from 10^−1^ to 10^−10^. Fig 5A illustrates the significance of the signal observed for both Polε and Msh2 at *ARS1207* and *ARS1209*, whereas no significant signal is seen at the adjacent inactive *ARS1208*. Additionally, *ARS1213* and *ARS416* display overlapping, highly significant signal from Polε and Msh2 (Fig 5B and 5C).

In summary, the Mcm4 signal is highly significant and is observed at each potential origin of replication. During S phase, Msh2 and Polε signal are co-incident in the majority of the active origins and flanking regions with high significance. These data confirm that Msh2, the invariant member of the mismatch recognition complex, remains closely associated with the replisome throughout S-phase of the cell cycle.

### The mismatch recognition complex displays an altered pattern of binding during replication in strains expressing mismatch repair defective PCNA/Pol30 variants

Having determined that the mismatch recognition complex is closely associated with the replisome throughout S phase, we wanted to explore what factors might influence MMR protein loading at origins as well as efficient scanning of the genome during replication. PCNA plays a critical role in MMR at multiple stages [16, 17, 55, 56]. To determine whether PCNA mutants implicated in MMR alter the binding and movement of the MMR recognition complexes during S phase, we examined Msh2 and Polε dynamics in PCNA mismatch repair defective strains.

**Fig 6:**
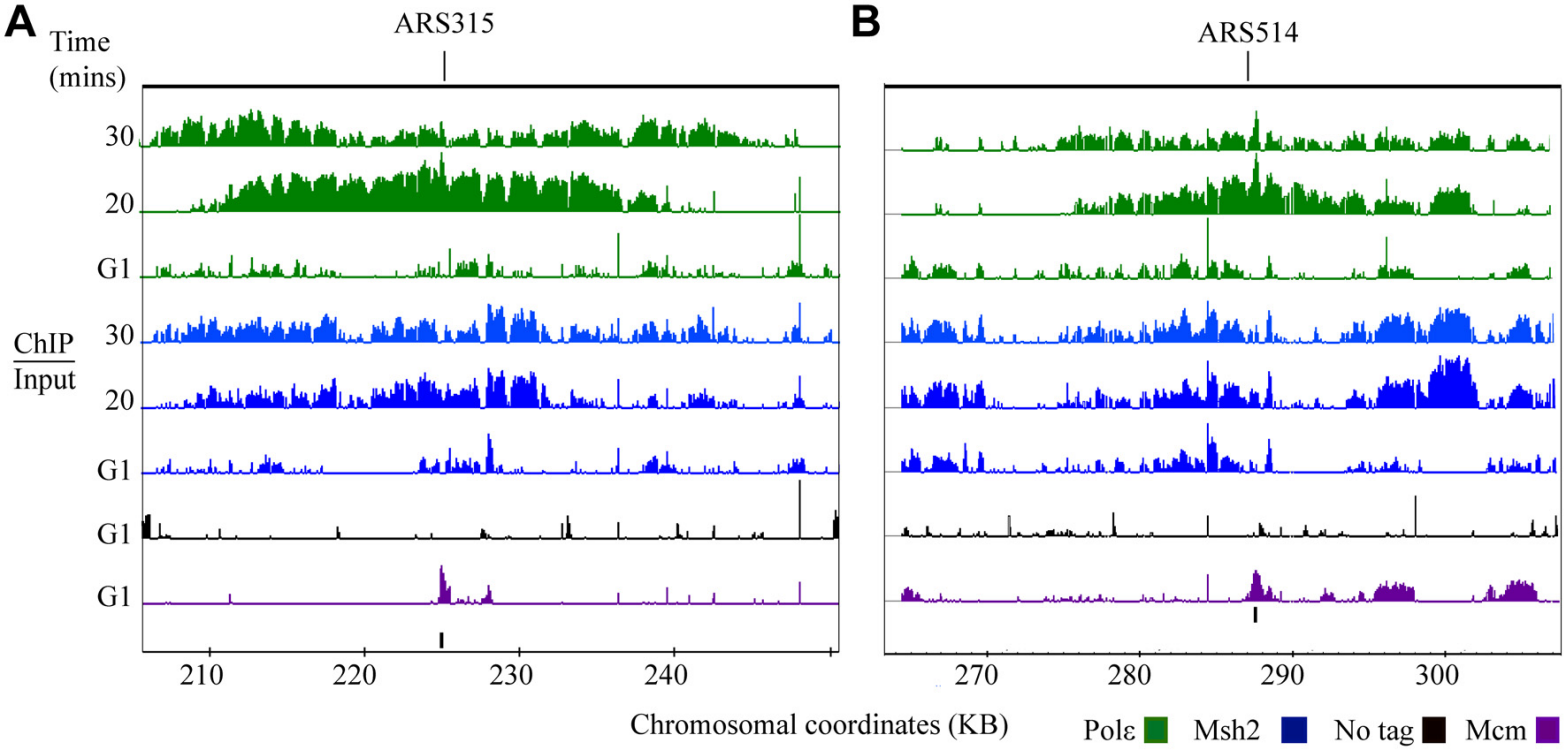
Msh2 and Polε co-localize to origins during S phase in a strain expressing a PCNA/Pol30 MMR defective variant, Pol30^C22Y^. The samples were analyzed at the time of arrest (G1) and two additional time points in S phase, 10 minutes apart (20 min and 30 min) for Polε and Msh2. The log_2_ (ChIP/Input) were visualized as using the Integrated Genome Browser for Mcm4 (Mcm, purple), no tag (black), Msh2 (blue) and Pol2 (Polε, green). The graphs were set to 2.5 for all data (~6 fold maximum increase). Black bars below the data denote position of origins in the genome databases. Chromosomal coordinates represent x10^3^ kb. Representative regions are shown including: **(A)** *ARS315* **(B)** *ARS51*.

Two missense variants of yeast PCNA (Pol30) were previously reported to disrupt MMR, but not to alter replication significantly [56]. We reasoned that the “separation of function” variants would be good candidates for determining the role of PCNA in mismatch recognition complex dynamics during replication. We first utilized the *pol30-201* separation of function mutant coding for Pol30^C22Y^ in ChIP-chip experiments. Pol30^C22Y^ confers a partial MMR defect; however, the Pol30^C22Y^ variant still interacts with MutSα (Msh2/Msh6) *in vitro* [56]. In this strain, the Polε signal appears normally distributed (relative to strains expressing wild-type PCNA/Pol30), suggesting that there is no effect on the processivity of the polymerase (Fig 6A and 6B). Cell cycle progression is also unaffected in this mutant, further supporting the absence of a replication defect [56]. The Msh2 signal coincided with Polε signal in the presence of the MMR defective Pol30^C22Y^ variant (Fig 6). This is in agreement with *in vitro* studies that show interaction is not fully disrupted between MutSα complexes and Pol30^C22Y^ protein [56]. However, the Msh2 signal does seem reduced in some regions adjacent to the origins (right side of *ARS315*, Fig 6A), suggesting a potential defect in association.

**Fig 7:**
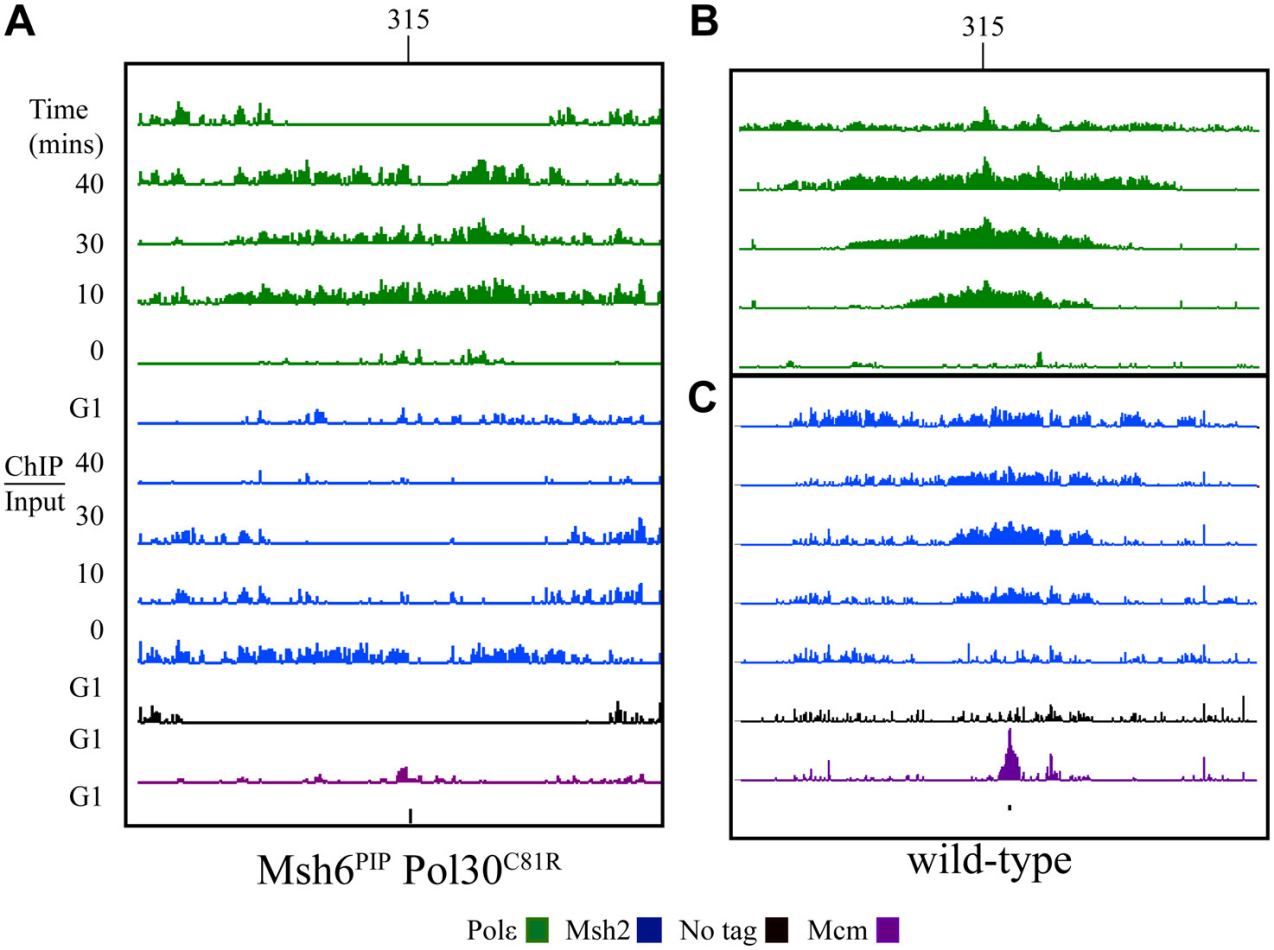
Msh2 displays aberrant binding at origins in a double mutant strain that disrupts the interaction between MutSα and PCNA. **(A)** Double mutant cells (*msh6-F33A*,*F34A pol30-204*) expressing the Msh6^PIP^ and Pol30^C81R^ were analyzed at the time of arrest (G1) and additional time points 10 minutes apart during S phase for Polε and Msh2. The log_2_ (ChIP/Input) were visualized as using the Integrated Genome Browser for Mcm4 (purple), no tag (black), Msh2 (blue) and Polε (green). The graphs were set to 2.0 for all data (~6 fold maximum increase). The black bar below the data denotes the position of *ARS315*. The same region for wild-type is shown for comparison for Pol2 (B) and Msh2 (C). The images in panes B and C are also shown in Figs 1 and 2.

Previous work showed that the partial MMR defects caused by the separation of function variants Pol30^C22Y^ and Pol30^C81R^ are exacerbated by converting two conserved phenylalanine residues to alanines in the PCNA interacting region (PIP box) of Msh6 [56]. Additionally, strains expressing Pol30^C81R^ have a partial MMR defect that is more severe than is seen in strains expressing Pol30^C22Y^ [56]. Finally, in strains expressing Pol30^C81R^ in combination with the Msh6 PIP box variant (Msh6^PIP^), MutSα no longer associates with replication foci [28]. We engineered strains containing *pol30-204* (coding for Pol30^C81R^) and the *msh6-F33A*,*F34A* PIP box mutation (expressing Msh6^PIP^) to analyze mismatch recognition complex dynamics during replication. Consistent with the finding that Pol30^C81R^ does not affect replication, the engineered strain exhibited normal cell cycle progression (S7 Fig). The ChIP-chip data shows some Msh2 signal in the vicinity of the replisome in the strain expressing Pol30^C81R^ and Msh6^PIP^ (Fig 7A); however the signal is not highly correlated with the Polε signal. Fig 7B and 7C show a comparative example of the typical signal for each protein in wild-type cells.

Two explanations could account for the presence of Msh2 signal in a strain in which the interaction between PCNA and MutSα should be diminished. First, the mismatch recognition complex might have alternative mechanisms for loading at origins as has been described previously [28, 57]. Second, the signal may be from MutSβ, which is known to be partially redundant with MutSα. The second explanation is in contrast to the studies showing that MutSβ does not co-localize with the leading strand polymerase during replication [28]. However, studies using human cell lines have also identified MutSβ at sites of active replication [27]. In the following sections, we address the issue as to whether both MutS complexes are needed for mismatch recognition and whether they are both found at origins during replication.

### MutSβ and MutSα are required for the full spectrum of mismatches generated during replication

To confirm on a genome-wide level that both MutS complexes are required for the full spectrum of mutations generated during replication, we performed mutation accumulation experiments followed by whole genome sequencing in strains lacking one of the components of the two mismatch recognition complexes (Table 2). Wild-type, *msh2*, *msh6* and *msh3* knockout strains were propagated in rich medium (YEPD) for ~210 generations with bottlenecks every ~21 generations. Single isolates from each strain were propagated. We have included previously published [49] *msh2* null (*msh2Δ*) and wild-type data normalized to 210 generations for comparison. In this previous analysis, we determined that mutation rate for DNA mismatch repair null strains was ~1 mutation per genome per generation, 225-fold higher than the wild-type rate and that the mutation spectra for mismatch repair defective cells included insertions/deletions at homopolymeric runs(HPRs) (~87%) and at larger microsatellites (~6%), as well as transitions (~5%) and transversions (~2%) [49].

**Table 2:** Mutation accumulation in mismatch repair mutants over 210 generations.

Relevant genotype	Single nucleotide polymorphisms	Homopolymeric run insertion or deletion	Larger microsatellite insertion or deletion
MSH2*	1	0	0
*msh2*Δ*	15	177	8
*msh3*Δ^†^	0	5	6
*msh6*Δ^†^	14	3	0

NCBI SRA accession numbers: *SRP026313, †SRP057591

As was expected from the use of reporter constructs [58] mutation accumulation analysis of the *msh3*Δ strain revealed an increase in mutations at larger microsatellites at a rate comparable to a complete MMR knockout (*msh2Δ*). No single base substitutions and only a few single base insertion/deletions at homopolymeric runs were observed (Table 2). These data are consistent with having a fully functional MutSα (Msh2/Msh6), because MutSα is capable of repairing single base substitutions and single nucleotide indels in the absence of Msh3 [48]. The *msh6*Δ strain, acquired 14 single base substitutions. This observed number is also comparable to the single base substitutions observed in the MMR knockout (*msh2Δ*) strain. Of the 14 mutations observed 12 were transitions while 2 were tranversions, similar to the ratio of transitions to transversions observed for MMR defective cells [49 and references therein]. The *msh6*Δ strain accumulated 3 insertion/deletions at homopolymeric runs, whereas mutations were not observed at larger microsatellites, consistent with the repair of larger indels being MutSβ specific.

After ~210 generations, *msh2*Δ accumulated a large number of insertion/deletions at HPRs (177) relative to the single deletion of the binding partners. We observe only 5 and 3 insertion/deletions at HPRs in *msh3*Δ and *msh6*Δ respectively (Table 2). This underscores the functional redundancy of MutSα and MutSβ for repair at HPRs and is in agreement with previous genetic analyses showing that MutSα and MutSβ are redundant for the repair of homopolymeric runs [48]. Additionally, analyses in mammalian systems also demonstrate MutSα/MutSβ redundancy in repair of indels at homopolymeric runs [59–64].

In summary, using mutation accumulation assays we showed on a genome-wide level that Msh6 and Msh3 are fully redundant for repair of single-base indels at homopolymers and that each MutS complex is needed to repair the entire spectrum of mismatches generated during replication. Given this data, it is reasonable to conclude that both MutSα and MutSβ are needed at the replisome to capture all of the types of mismatches as they emerge.

### The levels of the individual MutS subunits are such that MutSα and MutSβ should be found at equivalent levels

Although both MutS complexes are needed for the full spectrum of mismatches generated during replication, it is possible that MutSα was previously found to be the replisome associated mismatch recognition complex [28] because MutSα is more abundant. Previous studies have examined the levels of the MutS complexes in human [59, 65] and yeast cells [66]. In yeast, high throughput abundance studies reported ~ 1,000 copies of Msh2, ~5,000 of Msh6 and ~700 of Msh3 per cell [66]. The data suggest that MutSα accounts for a greater percentage of the MutS complexes present in the cell. However, the high throughput experiments in yeast required validation.

We examined the relative abundance of the individual components of both mismatch recognition complexes, using western blot analysis. We engineered a strain in which all three proteins were tagged with an identical myc epitope. The fusions were engineered at the endogenous chromosomal positions using the native promoters. The tagged proteins were shown to be functional for mismatch repair *in vivo*. The molecular weights of Msh2, Msh3, and Msh6 are sufficiently distinct to resolve the proteins on a 7% acrylamide gel. Although there may be differences in the accessibility of the multiple epitopes in each protein, this method provides a more direct comparison of the relative abundance of singly tagged strains.

**Fig 8:**
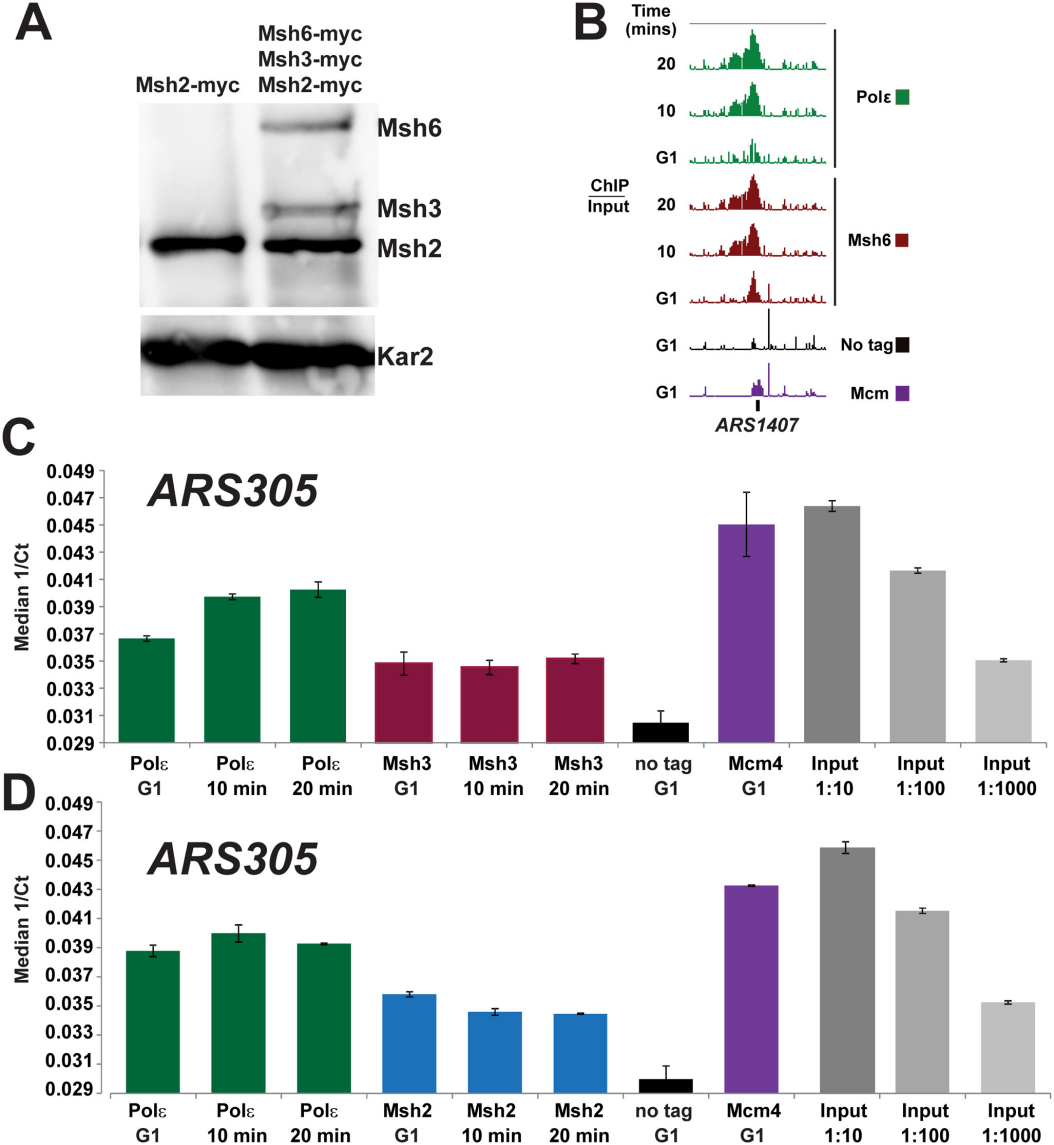
MutSα and MutSβ both bind origins during replication. **(A) Msh2, Msh3, and Msh6 levels are consistent with equal ratios of MutSα and MutSβ in the cell**. Cultures were grown to mid-exponential phase and proteins were extracted and detected by immunoblotting. The proteins were detected using antibodies for the myc epitope. Lane 1: contains Msh2-myc tagged extracts. Lane 2: all three components of the MutS complexes are myc-tagged (Msh2-myc, Msh6-myc, and Msh3-myc). The loading control was visualized using α-Kar2 antibody. The bands were quantified using image J software. **(B)** MutSα tracks with the replisome. Cells were processed for ChIP-chip as described above. An example of binding of Msh6 and Polε at *ARS1407* is shown. The log_2_ (ChIP/Input) were visualized as using the Integrated Genome Browser and the y-axis is set at 3 (or ~8 fold maximum) for each row. Msh6 (red-brown), Polε (green), no tag (black) and Mcm4 (purple) signals are included. **(C)** MutSβ binds *ARS305* during S Phase. Samples were prepared for ChIP as described above. The DNA was quantified by PCR (qPCR) to ensure that a ChIP-specific signal was detectable. Three technical replicates were performed for each time point. Samples were amplified and the threshold cycles (Ct) were determined using the Sequence Detection System, SDS version 2.3 software (Applied Biosystems). ChIP DNA samples for Polε (green), Msh3 (red-brown), no tag (black) and Mcm4 (purple) as well as input DNA at three dilutions were quantitied using pPCR. The error bars represent standard error of the mean. **(D)** Msh2 binding of *ARS305* during S Phase. Samples were prepared and analyzed using ChIP-PCR as described above for Panel C. ChIP DNA samples for Polε (green), Msh2 (blue), no tag (black) and Mcm4 (purple) as well as input DNA at three dilutions were quantitied. The error bars represent standard error of the mean.

The data reproducibly show that the relative abundance of Msh2:Msh6:Msh3 is 2:1:1 (Fig 8A). This ratio is visualized by examining the protein extract from the strain in which all three proteins are identically tagged (lane 2, Fig 8A). The discrepancy with our results and the high throughput method may be due to differences in the method of visualizing protein levels, the epitope tag used, or because the high throughput measured the levels from singly tagged strains. In summary, we find that Msh2 is in a 2-fold excess of the Msh6 and Msh3 binding partners such that there could be equal levels of MutSα and MutSβ in the cell; however, the experiments do not prove that the complexes are actually formed, that they are functional or that they are properly localized.

### Both MutSα and MutSβ are detected at origins during DNA replication

The data presented here and previously suggest that MutSα and MutSβ are needed to cover the full spectrum of mutations and the levels of the protein subunits suggest that the stoichiometry of the MutS complexes may be in balance; however it is still possible that MutSα is the major complex found at the replisome and that MutSβ only binds when larger indels form at the advancing fork. Additionally, some of the MutSβ complexes could be partitioned to function in other processes such recombination [67]. We therefore aimed to determine whether both complexes associate with the replisome during replication.

The tracking expreriments presented above examined Msh2 dynamics and therefore do not allow for the determination of whether one or both mismatch recognition complexes are co-incident with the polymerase throughout S-phase. To determine if MutSα (Msh6/Msh2) and MutSβ (Msh3/Msh2) are both in the vicinity of the replisome throughout S-phase, we performed ChIP-chip time course experiments using strains with Pol2-HA tagged (Polε) and either Msh6-myc tagged (MutSα) or Msh3-myc tagged (MutSβ).

A simplified time course time course experiment was performed to examine MutSα or MutSβ binding during S-phase. Briefly, samples were taken at the time of arrest and two additional times during S phase of the cell cycle and processed for ChIP. The samples were analyzed with the custom tiling arrays for Msh6 and using quantitative PCR (ChIP-PCR) for Msh3.

Fig 8B shows an example of the data for MutSα during replication. As was observed for Msh2, we observed binding of Msh6 in regions corresponding to Polε binding (~90% co-occupancy for 55 origins).

Using qPCR to detect binding to *ARS305*, an early firing origin with a robust signal (Figs 1 and 2). We observed that Msh3 is enriched at the origin with a signal similar to Msh2 (Fig 8D). The Msh3 signal is significantly higher than is observed in the no-tag control. Additionally, as is seen routinely with Msh2 ChIP-chip (Fig 2A) and ChIP-qPCR (Fig 8D), the Msh3 signal is not as strong as the Polε signal (Fig 8C and 8D). While this ChIP-qPCR approach does not show what occurs across the genome, it does provide an example of a highly efficient, early origin with Msh3-myc signal. In summary, we provide data consistent with a hypothesis positing that both MutSα and MutSβ associate with the replisome to capture the entire spectrum of mismatches that escape DNA polymerase proofreading.

## Discussion

Using high resolution genomic methods we determined that Msh2 tracks with the replisome throughout DNA replication. The Msh2 signal was distributed in the region occupied by the leading strand DNA polymerase and appeared to persist after fork passage. Additionally, we established that on a genome-wide level that both MutSα and MutSβ are required to efficiently repair single base pair substitutions (MutSα), single base indels at homopolymers (MutSα and MutSβ) and larger indels at microsatellites (MutSβ). Additionally, we examined the levels of the individual components of the MutSα and MutSβ. We observed Msh2 protein levels, in excess of Msh3 and Msh6, which both occur at equivalent levels. Finally we determined that MutSα and MutSβ are both detected at the replisome during S phase. These findings support the model that the mismatch repair recognition complex remains in close proximity to the errors as they emerge from the replisome as well as to the replication-specific nicked DNA that serve as strand specificity signals.

### Both MutSα and MutSβ are detected at origins during DNA replication to detect the full spectrum of mismatches emerging from the replisome

Our findings show that both MutSα and MutSβ are detected at origins during DNA replication. This finding contradicts the studies showing that MutSβ does not co-localize with the leading strand polymerase using fluorescence microscopy as the method of detecting associations [28]. Many reasons could account for the difference, including differences in detection methods. We are not able to detect the relative amounts of the complexes at the origins and it is possible that MutSα is more abundant and easily detected by both methods. Our finding that both complexes are present is consistent with studies using human cell lines where hMSH2, hMSH3 and hMSH6 are found at sites of active replication [27]. Additionally, using mass spectrometry, MutSβ was shown to interact with the replisome in *Schizosaccharomyces pombe* (Karin McDonald and Virginia Zakian, personal communications). Taken together, we favor a model where both complexes track with the replisome to cover the full spectrum of mismatches generated during replication.

### Potential role for PCNA in mismatch recognition complex loading during replication

In this work we showed that the mismatch repair complex loads at origins of replication with kinetics similar to the DNA polymerase during S phase. The precise mechanism of loading at origin is not known. One hypothesis we explored was that PCNA was responsible for the loading and potentially for aiding in the scanning efficiency. We found that the mismatch recognition complex signal is still observed in the presence of the PCNA variants that perturb the interaction with MutSα however, the signal was aberrant in the mutant strains. These data are consistent with a model in which there is a PCNA independent association of the mismatch recognition complexes with the replisome. In this model, PCNA plays an important role in MutSα/β dynamics during replication, but it is not the sole determinant controlling MutSα/β loading at origins. This model is supported by findings showing a PCNA dependent and independent mechanism for mismatch repair [28, 57].

### A model for efficient mismatch scanning of newly replicated DNA

Using chromatin immunoprecipitation and DNA tiling arrays, we are able to visualize the dynamics of MutSα/β binding during S phase; however, two models for movement along the DNA are consistent with the data: (1) MutSα/β loads at origins and scans immediately behind the advancing replisome facilitated by direct interactions with replisome components, or (2) MutSα/β loads at origins, but scans independently of the replisome. Because the two models involve loading of MutSα/β at active origins where the chromatin has been cleared, they both address the protein blockage problems discussed earlier.

The first model is dependent upon a physical connection between MutSα/β and the replisome. Live-cell imaging during S-phase of *S*. *cerevisiae* cells show that Msh6 co-localizes with Pol2 [28]. Additionally, as mentioned above, MutSα/β has been shown to interact with PCNA [17, 18, 30, 55, 68] and PCNA is associated with the replisome [69]. Finally, in *Bacillus subtilis* MutS and MutL have been shown to interact with the catalytic subunit of the DNA polymerase III (DnaE) *in vitro* [70]. *In vivo* experiments in *B*. *subtilis* using GFP-tagged DnaE showed that mismatch detection causes the polymerase to disengage from the DNA during replication [70]. These experiments support the model that MutS and MutSα/β are directly associated with the replisome. Thus, we favor the first model based on the previous studies and our observations that the distribution of Msh2 signal is very similar to the distribution of the leading strand DNA polymerase as the replisome advances during S phase. The first model is also appealing because tracking directly behind the replisome ensures that the MutSα/β complexes are always in close proximity to a strand specificity signal: the 3’-OH of the newly synthesized strand.

A further refinement of the model includes the following: MutSα/β loads at origins and scans immediately behind the advancing replisome as well as in the regions behind the replication fork. The addition to the model is based on the fact that the MutSα/β signal persists in the newly replicated region even when the leading strand polymerase appears to have cleared the region. The persistence of signal could be explained by the interaction of MutSα/β and PCNA. In eukaryotes, PCNA is known to accumulate behind the replisome [71] and in *B*. *subtilis*, DnaN (PCNA) clamp zones have been shown to remain behind the replication zone [72]. This DnaN-mediated recruitment of MutS is responsible for 90% of repair in *B*. *subtilis* with the remaining mismatch repair being DnaN-independent [32]. Taken together, we favor a model in which the persistent MutSα/β signal after fork passage is explained, in part, by interactions with PCNA molecules that remain behind the replisome.

**Fig 9:**
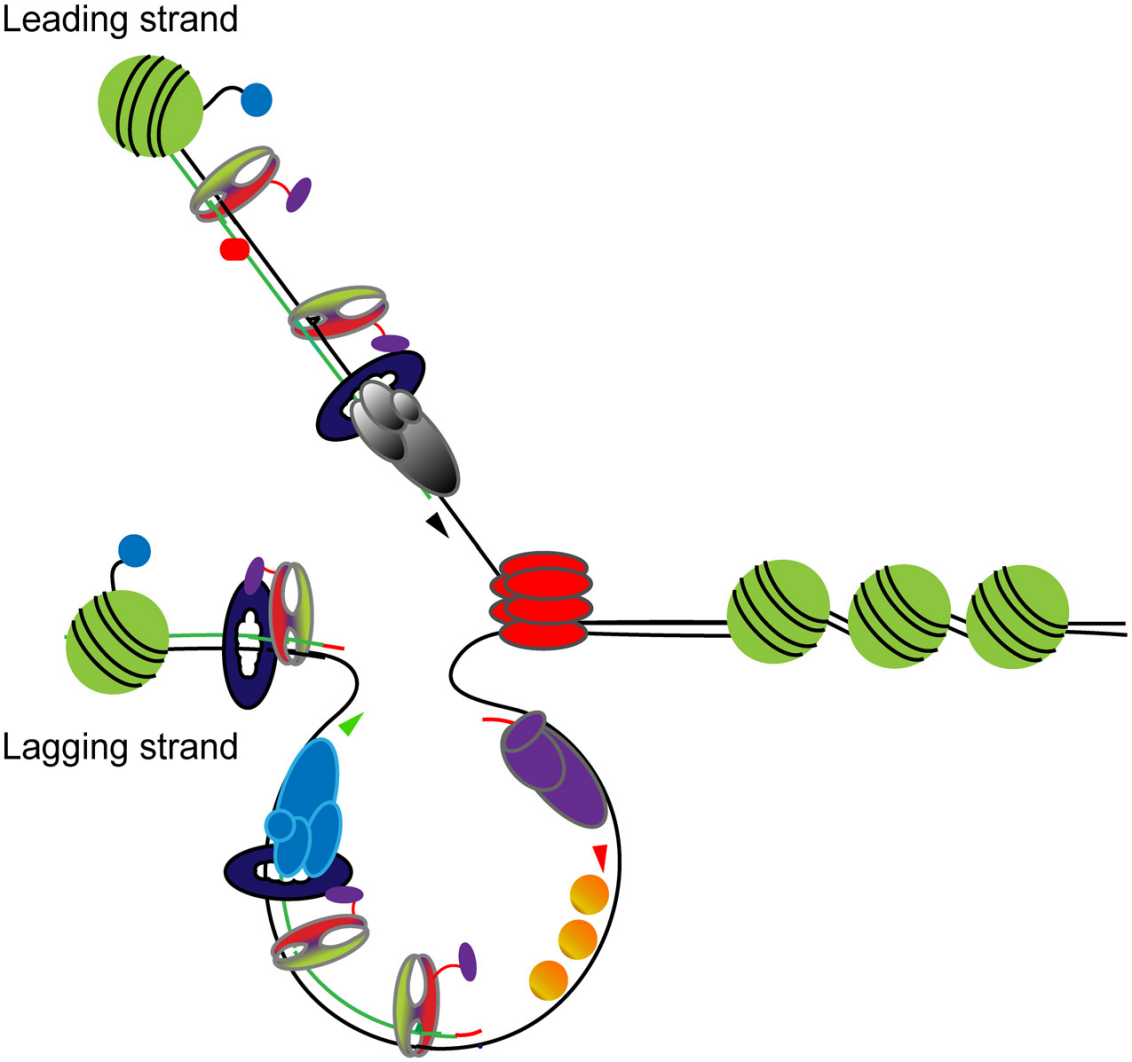
Model for MutS mismatch recognition during replication. The model described in the text is depicted above with schematics of MutS complexes (green and red) with a flexible tether (purple); DNA polymerase (multi-subunit complexes shown in light blue and grey); PCNA (dark blue circles), unmodified histones (green circles); modified histones (green circles with blue tag); single stranded binding proteins (orange circles); DNA polymerase alpha (multi-subunit complex shown in purple circles); and the Mcm4 helicase (red). The template DNA is shown with black lines and the newly synthesized DNA with green lines. The direction of polymerization of the DNA is shown with arrowheads.

Fig 9 illustrates a model for MutSα/β signal distribution during replication. In the model, MutSα/β complexes bind to activated origins during S phase with a timing similar to DNA polymerase. The MutSα/β loading may be facilitated by direct PCNA interactions or modified histones may function to recruit MutSα/β to active origins. Once MutSα/β is loaded, a close association with the advancing replisome ensures that mismatches are rapidly detected and that the MMR machinery always has a proximal replication-specific nick to direct repair to the newly synthesizes strand. In the model, upon detection of a mismatch, the most proximal signal is the 3’-OH of the newly synthesized strand. The MutSα/β signal persisting behind the advancing replisome may be a consequence of PCNA interactions. PCNA is bound to nicks behind the replisome created during lagging strand synthesis and caused by rNMP excision.

## Materials and Methods

### Microbial manipulations and molecular techniques

Yeast strains used in this work are listed in S3 Table. Microbial and molecular manipulations were conducted according to previously published procedures [73, 74]. Plasmid DNA extractions were performed using the Qiagen procedure (Qiagen Inc., Valencia, CA). Primers were synthesized by Integrated DNA Technologies Inc. (Coralville, IA). Restriction endonuclease digestions and polymerase chain reactions (PCR) were performed using the enzyme manufacturer recommended reaction conditions (New England Biolabs, Beverly, MA).

### Strain constructions

The *MSH2-myc POL2-HA* strain was constructed by a genetic cross using strains from the Gammie laboratory (*MSH2-myc*) and the Bell laboratory (*POL2-HA*). The *pol30* mutant strains were constructed by creating the mutations on a centromere-based plasmid by recombination [75] and cloning the mutated *pol30* gene into a *URA3*-based integrative plasmid vector backbone [76]. The mutations were introduced into the chromosome by a two-step integration method [77]. To produce the Msh6 PIP box mutant (*msh6-F33A*, *F34A*) we employed *in vivo* site directed mutagenesis [78]. We sequenced the *MSH6* locus and confirmed the change resulting in replacement of the two conserved phenylalanines at codons 33 and 34 with alanines in the PIP box of *MSH6*.

To chromosomally tag *MSH6* and *MSH3* at the C-terminal coding regions, the myc or HA epitope tag and the kanamycin gene was amplified from the pFA6-x13myc or pFA6x3HA plasmids as described previously [79]. PCR amplified products were transformed into wild-type W303. Integration was confirmed by PCR amplification of the epitope tag and sequencing. Western blot analysis was employed to confirm expression of the tag. Finally, the functionality of the fusions were confirmed by performing mismatch repair assays [80].

### Synchronization of the cell cycle

To achieve synchrony, cultures are grown to mid-exponential phase (~0.5 OD_600_) in SC medium at 30°C. The cells were then shifted to 18°C to slow the growth rate and arrested in the G1 phase of the cell cycle with 10 μg/ml α–factor. The cells were released from G1 arrest by washing the cells and resuspending in fresh medium. Samples were taken initially at 6 or 10-minute intervals for ChIP-chip, followed by 30 minute intervals for continued analysis of DNA content. Samples were collected for each time-point; the cells were cross-linked with freshly made 4% para-formaldehyde (final concentration ~1%) and flash frozen in liquid nitrogen. Aliquots from each time point were processed and analyzed by flow cytometry to determine which samples correspond to the cells in S phase.

### Chromatin immunoprecipitation

An aliquot of the fixed samples were processed for flow cytometry as previously described with modifications [81]. Briefly, cells were incubated with RNAase and SYTOX Green and the DNA content per cell was measured using the Becton-Dickinson LSRII Multi laser analyzer. Samples corresponding to S-phase of the cell cycle were then processed for ChIP-chip. The samples were processed for ChIP by mechanically disrupting the cell walls using a Fastprep -24 instrument (MP Biomedicals LLC) followed by sonication to generate DNA fragments averaging ~500 bp (Covaris S220 Focused-ultrasonicator). A portion of each sample was retained as the input DNA. The remaining sample was split into two equal fractions and the cross-linked protein/DNA complexes were immunoprecipitated with antibodies conjugated to agarose beads (one fraction with α-HA for Polε and the other with α-myc for MutSα/β complexes). To obtain signal corresponding to both MutSα/β and Polε, fixation conditions required optimization. Fixation for 45 minutes with freshly prepared para-formaldehyde facilitated the immunoprecipitation of both proteins. To confirm that the Msh2-myc tagged mismatch repair protein was immunoprecipitated, a time point in S phase was collected in duplicate. The sample was processed using identical conditions and the crosslinks were reversed. Western blot analysis was performed as described previously [82] to verify the immunoprecipitations.

For the time course experiments, the cross-links from the ChIPs and inputs were reversed and the DNA was purified. Routinely, a portion of the samples was quantified by PCR to ensure that a ChIP-specific signal was detectable using Power SYBR Green PCR master mix (Applied biosystems). Three technical replicates were performed for each time point. Samples were amplified for and the threshold cycles (Ct) were determined using the Sequence Detection System, SDS version 2.3 software (Applied Biosystems).

### Hybridization of custom tiling arrays

Both the input and ChIP DNA were amplified using ligation-mediated PCR [83] and labeled with fluorescent dyes Cy3 and Cy5 respectively (reverse dye labeling controls were also performed). Labeled samples were hybridized to custom DNA tiling arrays with 15,000 probes (Agilent technologies). The 24 regions represented include early, middle, and late firing origins and at least 20 kb of flanking DNA. A total of 65 origins were represented on the arrays: 53 are confirmed origins, 3 have previously been identified as likely origins, 6 proposed origins and 3 as dubious origins [36]. Additional features included telomere sequences, silenced loci, tRNA genes, highly transcribed genes and long terminal repeats, which are known replication pause sites [84]. Additionally, mono-, di- and tri-nucleotide repeats were included because these regions are associated with insertion/deletion loops requiring mismatch repair [85].

### ChIP data analysis

An Agilent DNA microarray scanner was used to detect the fluorescence intensities for Cy3 and Cy5. The data was subsequently processed using Agilent Feature Extraction Software. Algorithims were used to correct for background and normalize the data and the log_2_ ratios of ChIP/input were calculated for the adjusted data. The data were then uploaded into the Princeton University microarray database (PUMAdb). PUMAdb has features that facilitate data visualization and processing for a variety of programs. The data files processed in PUMAdb were converted to files compatible with Integrated Genome Browser (IGB) (Affymetrix). IGB was used to represent the data as log_2_ ratios for individual experiments (Version 8.0.1)[86].

For replicate experiments we used the Chipper software [54] with minor modifications. Chipper analysis generates the significance (*p*-values) of enrichment obtained from individual experiments using variance stabilization and not log_2_ ratios. The averaged data were visualized using IGB. The data are provided in an attached supplement (S1 data).

### Fork progression analysis

To determine the rate of fork progression the leading edge of the peak for each time point was measured. The difference the time points (tp) were then taken and divided by the time interval (*Rate of fork progression =* (*tp* ^*1*^ *- tp* ^*2*^)*/ time interval*). For two experiment sets, the average rate of fork progression of ~423 bp/min and ~ 438 kb/min respectively was determined for all origins by analyzing the bi-directional movement. In a few instances the leading edge was not discernable due to background signal.

### Mutation accumulation

For the mutation accumulation and whole genome sequencing, the wild-type, *msh2*, *msh6* and *msh3* knockout strains were propagated in rich medium (YEPD) for ~210 generations with bottlenecks every ~21 generations. Genomic DNA preparations, whole genome sequencing, and data analyses were as described previously [49].

### Quantitative PCR

Quantification of DNA enrichment in the ChIP and the input was performed using Q-PCR (Power SYBR Green PCR master mix, Applied biosystems). Three technical replicates were performed for each time point. Samples were amplified for and the threshold cycles (Ct) were determined using the Sequence Detection System, SDS version 2.3 software (Applied Biosystems). For the amplification of *ARS305*, the forward and reverse primers used were 5’- GATTGAGGCCACAGCAAGAC-3’ and 5’- TCACACCGGACAGTACATGA-3’ respectively.

## Supporting Information

S1 Fig Experimental design and flow cytometry. **(A)** Outline of the experimental design for cell synchrony. (**B)** The flow cytometry data shown are representative of the cell cycle arrest and synchrony for the ChIP-chip experiments. Cells were arrested in G1 with α-factor at 18°C. The cells were washed twice to remove α-factor, resuspended in fresh medium and returned to 18°C. Samples were removed at the indicated time points and analyzed by Becton-Dickinson LSII Multi laser analyzer. The amount of SYTOX Green bound to DNA was measured by flow cytometry analysis. The data are shown in the graph where the x-axis represents DNA content per cell (haploid, 1N and diploid, 2N), the z-axis represents time points (min) after release from arrest and the y-axis denotes cell count. A total of 100,000 cells were collected for each time. The samples used in the ChIP-chip analysis are indicated, including 0 min and 108–144 min corresponding to complete G1 arrest and S-phase of the cell cycle respectively.
(PDF)

S2 Fig Confirmation of chromatin shearing. Formaldehyde fixed samples were sonicated to shear chromatin and crosslinked proteins (Msh2-myc and Pol2-HA) were immunoprecipitated (IP). After crosslink reversal, 5 μl of each IP were run on a 1.5% agarose gel stained with SYBR safe. The image is representative of the size fragments generated. Each lane is a single time point for each IP.
(PDF)

S3 Fig Mcm4 binds both active and inactive origins of replication. The tiling array data were visualized using the Integrated Genome Browser, IGB, program (Affymetrix) and are depicted as peaks correspond to log2 ratios (ChIP/Input). The y-axis is set at 3 (or a ~8-fold maximum signal). Mcm4 signal is purple and the no tag control for non-specific binding is depicted in black. Black bars below the data denote position of *ARSs* in the genome database. Chromosomal coordinates represent X 10^3^ kb. Origins bound by Mcm4 helicase: active origins (*ARS305* and *ARS306*) and adjacent inactive origins (*ARS301*, *ARS303*, *ARS304* and *ARS320*).
(PDF)

S4 Fig Reproducibility of Polε distribution. Three independently performed experiments are depicted. Each row corresponds to ChIP-chip signal at the indicated times at G1 or to a time point series taken during S phase (0–5). The tiling array data were visualized using the Integrated Genome Browser program (Affymetrix) and are depicted as peaks correspond to log2 ratios (ChIP/Input). For each experiment set the y-axis is set at 3 (or ~8-fold maximum). Chromosomal coordinates represent X 10^3^ kb. The region corresponds to chromosome XVI which includes *ARS1619* and *ARS1633*.
(PDF)

S5 Fig Cell synchrony analysis of the strain expressing the Msh6^PIP^ variant and Pol30^C81R^. Double mutant cells (*msh6-F33A*,*F34A pol30-204*) expressing the Msh6^PIP^ variant and Pol30^C81R^ were arrested in G1 with **α**-factor to synchronize the cells. The cells were released from arrest and time points were taken every 10 min starting 90 min after release. The cells were fixed and a portion was prepared for flow cytometry analysis. The data indicate the DNA content per cell for unreplicated DNA content per cell (1N) and replicated DNA before cell division (2N). The results from the flow cytometry are shown for **(A)** the full time course and **(B)** for the G1 and S-phase samples and a few additional time points.
(PDF)

S1 Table Autonomously replicating sequences (*ARS*) on tiling array with Mcm4 binding. The table quantifies the binding of Mcm4 at the origins on the custom tiling array and provides the references for the original characterization of origin activity.
(PDF)

S2 Table Polymerase ε progression rates from two trials. The calculated base pairs per minute progression of Pol2 at select origins from two trials is given.
(PDF)

S3 Table Yeast strains used in this study. The yeast strains genotypes and sources are listed in the table.
(PDF)

S1 Data ChIP-chip data. The data corresponding to each figure are in this compressed file.
(ZIP)
