# Supplementary figures and images for: The Eukaryotic Mismatch Recognition Complexes Track with the Replisome during DNA Synthesis
Source: PLoS Genet. 2015 Dec 18;11(12):e1005719. doi: 10.1371/journal.pgen.1005719 (PMC4684283; doi:10.1371/journal.pgen.1005719)

**A**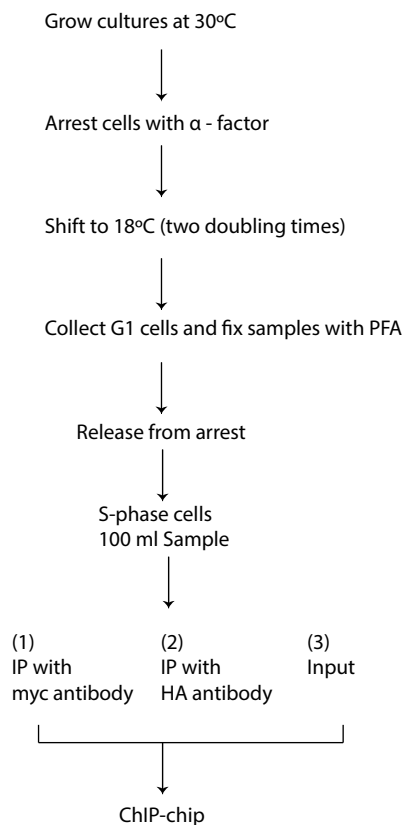**B**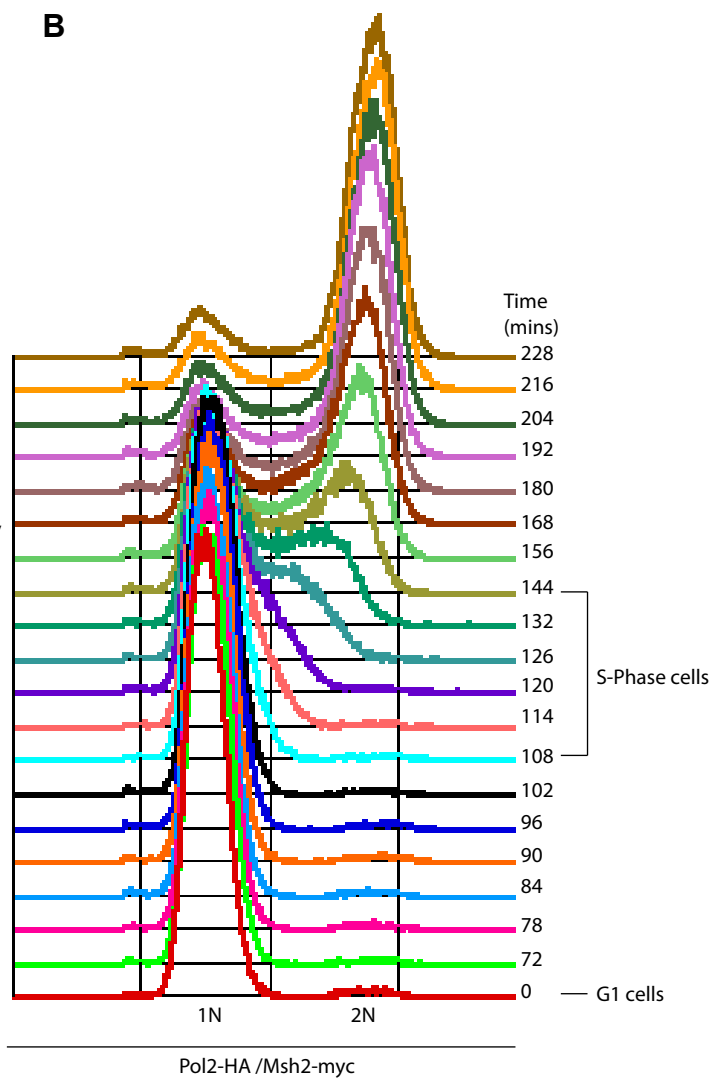

Supplement: S1 Fig — (A) Outline of the experimental design for cell synchrony. (B) The flow cytometry data shown are representative of the cell cycle arrest and synchrony for the ChIP-chip experiments. Cells were arrested in G1 with α-factor at 18°C. The cells were washed twice to remove α-factor, resuspended in fresh medium and returned to 18°C. Samples were removed at the indicated time points and analyzed by Becton-Dickinson LSII Multi laser analyzer. The amount of SYTOX Green bound to DNA was measured by flow cytometry analysis. The data are shown in the graph where the x-axis represents DNA content per cell (haploid, 1N and diploid, 2N), the z-axis represents time points (min) after release from arrest and the y-axis denotes cell count. A total of 100,000 cells were collected for each time. The samples used in the ChIP-chip analysis are indicated, including 0 min and 108–144 min corresponding to complete G1 arrest and S-phase of the cell cycle respectively. (PDF) [file pgen.1005719.s001.pdf]

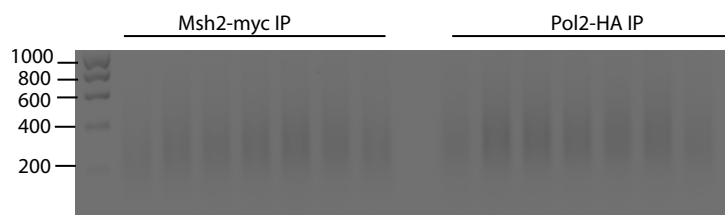

Supplement: S2 Fig — Formaldehyde fixed samples were sonicated to shear chromatin and crosslinked proteins (Msh2-myc and Pol2-HA) were immunoprecipitated (IP). After crosslink reversal, 5 μl of each IP were run on a 1.5% agarose gel stained with SYBR safe. The image is representative of the size fragments generated. Each lane is a single time point for each IP. (PDF) [file pgen.1005719.s002.pdf]

# Chromosome III

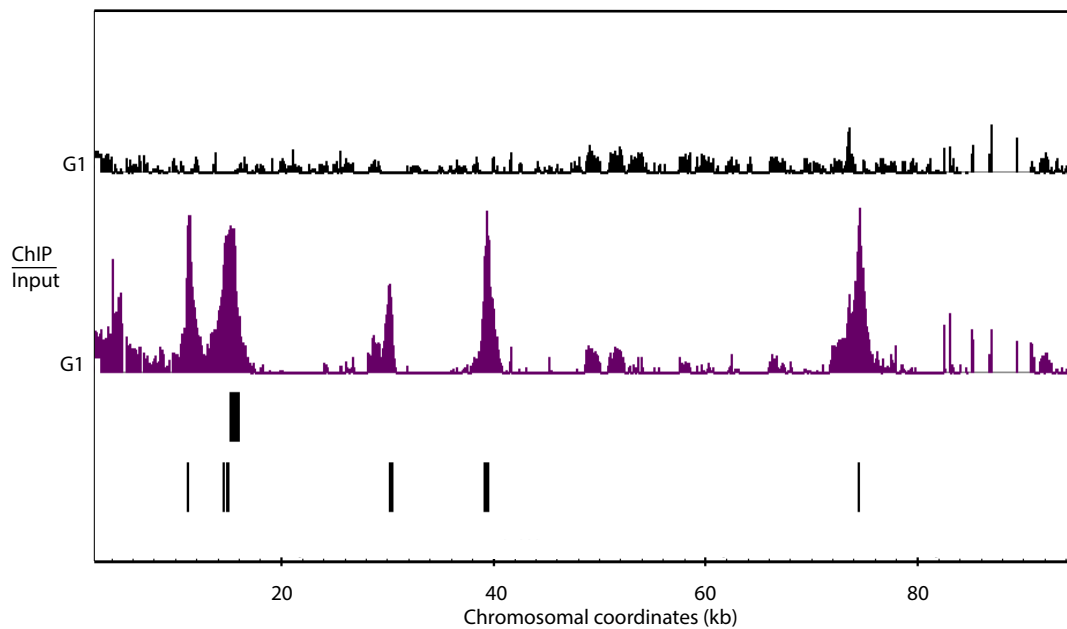

Inactive origins

(ARS301, ARS303, ARS304 and ARS320)

Active origins

(ARS305 and ARS306)

No tag  Mcm

Supplement: S3 Fig — The tiling array data were visualized using the Integrated Genome Browser, IGB, program (Affymetrix) and are depicted as peaks correspond to log2 ratios (ChIP/Input). The y-axis is set at 3 (or a ~8-fold maximum signal). Mcm4 signal is purple and the no tag control for non-specific binding is depicted in black. Black bars below the data denote position of ARSs in the genome database. Chromosomal coordinates represent X 103 kb. Origins bound by Mcm4 helicase: active origins (ARS305 and ARS306) and adjacent inactive origins (ARS301, ARS303, ARS304 and ARS320). (PDF) [file pgen.1005719.s003.pdf]

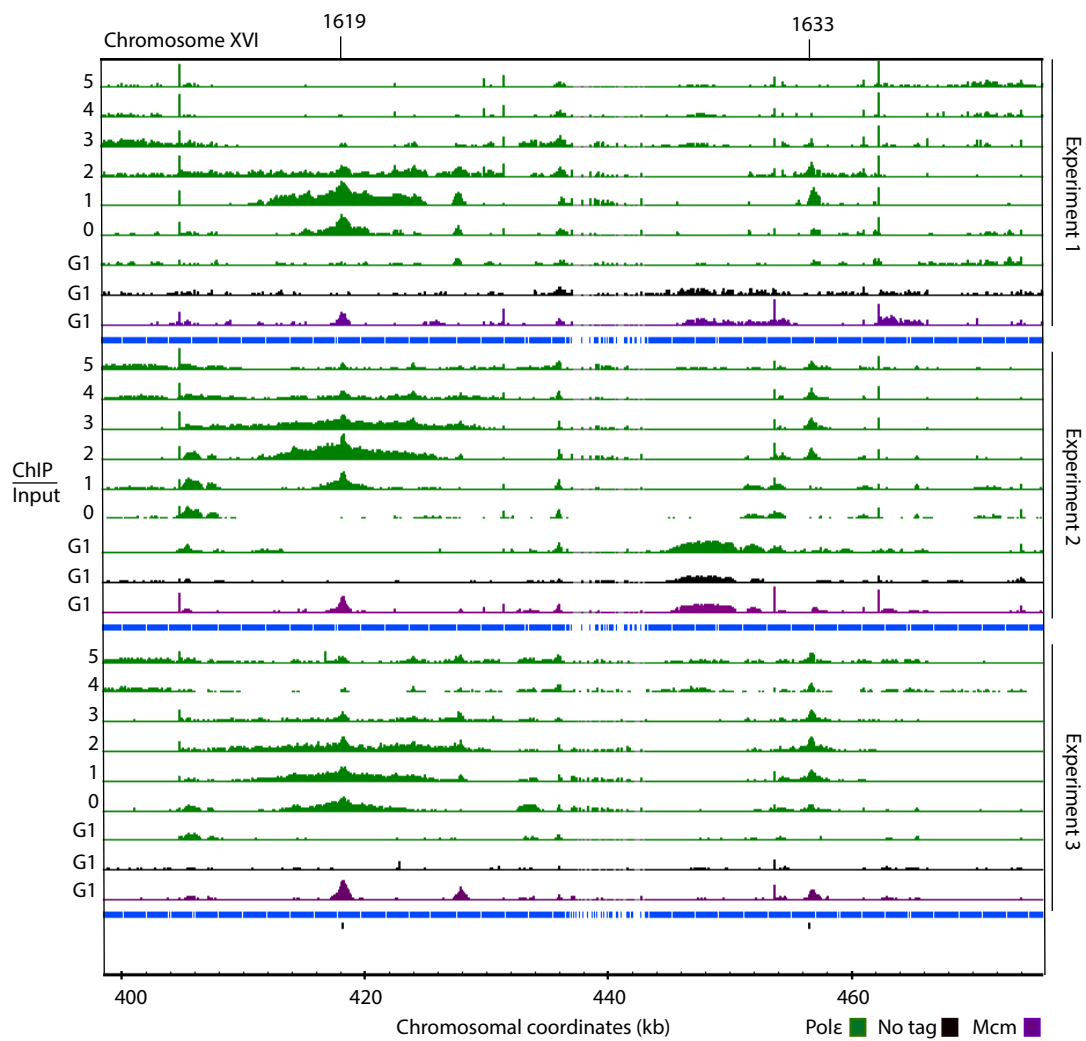

Supplement: S4 Fig — Three independently performed experiments are depicted. Each row corresponds to ChIP-chip signal at the indicated times at G1 or to a time point series taken during S phase (0–5). The tiling array data were visualized using the Integrated Genome Browser program (Affymetrix) and are depicted as peaks correspond to log2 ratios (ChIP/Input). For each experiment set the y-axis is set at 3 (or ~8-fold maximum). Chromosomal coordinates represent X 103 kb. The region corresponds to chromosome XVI which includes ARS1619 and ARS1633. (PDF) [file pgen.1005719.s004.pdf]

**A**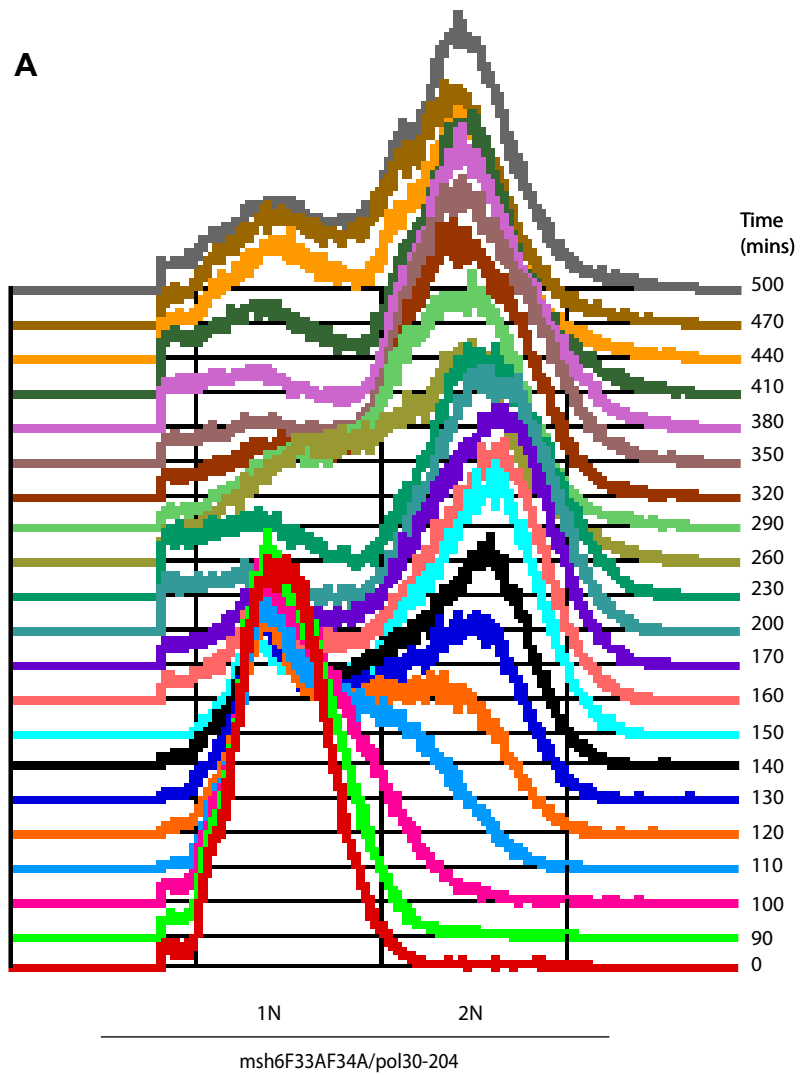**B**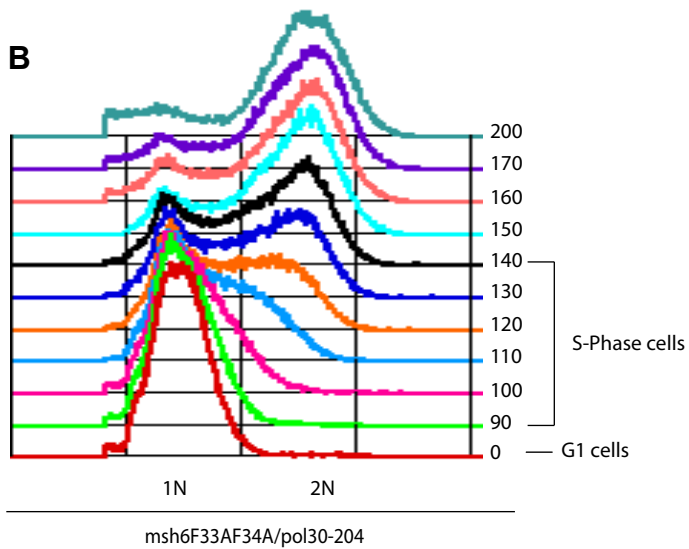

Supplement: S5 Fig — Double mutant cells (msh6-F33A,F34A pol30-204) expressing the Msh6PIP variant and Pol30C81R were arrested in G1 with α-factor to synchronize the cells. The cells were released from arrest and time points were taken every 10 min starting 90 min after release. The cells were fixed and a portion was prepared for flow cytometry analysis. The data indicate the DNA content per cell for unreplicated DNA content per cell (1N) and replicated DNA before cell division (2N). The results from the flow cytometry are shown for (A) the full time course and (B) for the G1 and S-phase samples and a few additional time points. (PDF) [file pgen.1005719.s005.pdf]
