# Supplementary material for: The Eukaryotic Mismatch Recognition Complexes Track with the Replisome during DNA Synthesis
Source: PLoS Genet. 2015 Dec 18;11(12):e1005719. doi: 10.1371/journal.pgen.1005719 (PMC4684283; doi:10.1371/journal.pgen.1005719)
Supplement: S1 Table — The table quantifies the binding of Mcm4 at the origins on the custom tiling array and provides the references for the original characterization of origin activity. (PDF) [file pgen.1005719.s006.pdf]

S1 Table. Autonomously Replicating Sequences (*ARS*) on Tiling Array with Mcm Binding

ARS	**Number of experiments Mcm loading observed**	**Other Microarray study identifying *ARS****
ARS104	5	a, c, d, e
ARS105	5	a-d
I-72$^{\psi}$	4	a, b
ARS216	5	a-d
ARS217	4	a, d
ARS300	4	c, d
ARS301	5	a, c, d
ARS302	3	a, c, d
ARS320	5	a, c, d
ARS303	5	a, c, d
ARS304	5	d
ARS305	5	a-e
ARS306	5	a, b, d, e
ARS308	5	b, d
ARS309	5	a-e
ARS315	5	a, b, d, e
ARS316	5	a-e
ARS317	4	a, c, e
ARS318	5	a, c, e
ARS319	4	d
ARS412	4	a, c, d
ARS413	5	a-e
ARS414	5	a-e
ARS415	4	a-e
ARS416	5	a-e
IV-477$^{\psi}$	1	a, d, e
ARS433	4	a-d
ARS514	5	c, d, e
ARS522	5	a-d
ARS523	5	a-d
ARS603	5	a-e
ARS607	5	a-e
ARS608	5	a, d
ARS609	5	a, c, d, e
ARS610	5	c, d
VII-17$^{\psi}$	5	a, b, d
ARS702	5	a-e
ARS919	5	a, c, d, e
ARS920	5	a-e
ARS1012	5	a-e
ARS1013	5	a-e
ARS1116	5	a-e

ARS	Number of experiments Mcm loading observed	Other Microarray study identifying *ARS**
ARS1117	4	a-e
ARS1207	5	a-e
ARS1208	5	b
ARS1209	5	a-e
ARS1210	3	b, c, d
ARS1211	5	a-e
ARS1211.5	3	a, b, d
ARS1213	4	a-e
ARS1407	5	a-e
ARS1412	5	a-d
XIV-180$^{\psi}$	1	b
ARS1517	5	a-d
ARS1518	2	a, c, d
ARS1519	5	a-e
ARS1520	4	a, c-e
ARS1619	5	a-e
ARS1621	5	a-e
ARS1624	4	a-d
ARS1625	4	a-d
ARS1633	5	a, b, d, e
XVI-428$^{\psi}$	3	d
XVI-553$^{\psi}$	4	a, b, d
ARS1634	5	a, b, d

* (a.) [1]; (b.) [2]; (c.) [3]; (d.) Xu et al. (2006)[4]; (e.) [5]. $^{\psi}$No ARS name has been assigned, chromosomal location obtained from OriDB [6].

References

1. Feng WY, Collingwood D, Boeck ME, Fox LA, Alvino GM, et al. (2006) Genomic mapping of single-stranded DNA in hydroxyurea-challenged yeasts identifies origins of replication. Nature Cell Biology 8: 148-U139.
2. Raghuraman MK, Winzeler EA, Collingwood D, Hunt S, Wodicka L, et al. (2001) Replication dynamics of the yeast genome. Science 294: 115-121.
3. Wyrick JJ, Aparicio JG, Chen T, Barnett JD, Jennings EG, et al. (2001) Genome-wide distribution of ORC and MCM proteins in S-cerevisiae: High-resolution mapping of replication origins. Science 294: 2357-2360.
4. Xu W, Aparicio JG, Aparicio OM, Tavare S (2006) Genome-wide mapping of ORC and Mcm2p binding sites on tiling arrays and identification of essential ARS consensus sequences in S-cerevisiae. Bmc Genomics 7.
5. Yabuki N, Terashima H, Kitada K (2002) Mapping of early firing origins on a replication profile of budding yeast. Genes to Cells 7: 781-789.
6. Siow CC, Nieduszynska SR, Mueller CA, Nieduszynski CA (2012) OriDB, the DNA replication origin database updated and extended. Nucleic Acids Research 40: D682-D686.
