# Supplementary material for: The Eukaryotic Mismatch Recognition Complexes Track with the Replisome during DNA Synthesis
Source: PLoS Genet. 2015 Dec 18;11(12):e1005719. doi: 10.1371/journal.pgen.1005719 (PMC4684283; doi:10.1371/journal.pgen.1005719)
Supplement: S2 Table — The calculated base pairs per minute progression of Pol2 at select origins from two trials is given. (PDF) [file pgen.1005719.s007.pdf]

S2 Table. Polymerase ε Progression Rates from Two Trials

Trial	*ARS*	Upstream Rate bp/min	Downstream Rate bp/min
1	*ARS1*	277	513
2		271	494
1	*ARS216*	353	416
2		324	442
1	*ARS305*	353	509
2		459	572
1	*ARS306*	551	-
2		544	-
1	*ARS309*	382	-
2		390	-
1	*ARS315*	303	302
2		379	251
1	*ARS413*	470	-
2		473	359
1	*ARS414*	432	-
2		433	-
1	*ARS415*	442	-
2		375	299
1	*ARS607*	475	512
2		466	499
1	*ARS919*	438	-
2		-	-
1	*ARS920*	498	448
2		-	445
1	*ARS1012*	404	443
2		400	455
1	*ARS1207*	559	324
2		566	345
1	*ARS1209*	273	448
2		285	-
1	*ARS1211*	551	491
2		487	-
1	*ARS1213*	570	508
2		522	417
1	*ARS1407*	310	414
2		315	464
1	*ARS1619*	565	544
2		571	485
1	*ARS1621*	-	-
2		389	334
1	*ARS1622*	-	-
2		478	-

Dash indicates that the value was not measurable
