# Supplementary material for: The Eukaryotic Mismatch Recognition Complexes Track with the Replisome during DNA Synthesis
Source: PLoS Genet. 2015 Dec 18;11(12):e1005719. doi: 10.1371/journal.pgen.1005719 (PMC4684283; doi:10.1371/journal.pgen.1005719)
Supplement: S3 Table — The yeast strains genotypes and sources are listed in the table. (PDF) [file pgen.1005719.s008.pdf]

**S3 Table. Yeast strains used in this study**

| Name | Genotype | Source |
|---|---|---|
| **MY10733** | *MAT***a** *MSH2-myc::KanMX ura3-1 leu2-3,112 can1-100 his3-11,15 bar1::HISG POL2-3xHA::LEU2* | Gammie laboratory |
| **MY10567** | *MAT***a** *MCM4-HA::TRP1 ade2-1 leu2-3,112 can1-100 his3-11,15 trp1-1 bar1::HISG* | Stephen Bell Laboratory, MIT |
| **MY15095** | *MAT***a** *POL2-3xHA::LEU2 MSH2-myc::KanMX pol30-204 leu2-3,112 his3-11,15 trp1-1 ura3-1 bar1::HISG can1-100 msh6-F33A,F34A* | Gammie laboratory |
| **MY11870** | *MAT***a** *POL2-3xHA::LEU2 MSH2-myc:: NatMX pol30-201 bar1 ura3-1 leu2-3,112 can1-100 his3-11,15 rad5-5* | Gammie laboratory |
| **AGY1198** | *MAT***a** *msh6Δ::HphMX ade2-1 trp1-1 ura3-1 leu2-3,112* | Gammie laboratory |
| **AGY1196** | *MAT***a** *msh3Δ::HphMX ade2-1 trp1-1 ura3-1 leu2-3,112* | Gammie laboratory |
| **MY14904** | *MAT***a** *MSH2-myc::KanMX MSH3-myc::KanMX MSH6-myc::KanMX ura3-1 his3-11,15 leu2-3,112* | Gammie laboratory |
| **MY13803** | *MAT***a** *POL2-3xHA::LEU2 MSH6-myc::KanMX can1-100 trp1-1 his3-11,15 bar1::HISG* | Gammie laboratory |
| **MY12327** | *MAT***a** *POL2-3xHA::LEU2 MSH3-myc::KanMX trp1-1 ura3-1 hom3-10 leu2-3,112 his3-11,15 bar1::HISG* | Gammie laboratory |

All strains are derived from W303 and confirmed to be wild-type (unless indicated) at the *RAD5* locus by PCR and at the *CAN1* locus by canavanine resistance assays.
